# Intrinsic Dual‐Phase Regulated GeSe_2_ Nanoparticles Triggered by Ball‐Milling Treatment for Photonic Multi‐Valued Logic Circuits

**DOI:** 10.1002/advs.76324

**Published:** 2026-07-01

**Authors:** An‐Ting Tsai, Chun‐Jen Wang, Pin‐Chao Liao, Chin Shan Lue, Chia‐Nung Kuo, Le Vo Phuong Thuan, Po‐Hsuan Hsiao, Chang‐Hyun Kim, Chia‐Yun Chen

**Affiliations:** ^1^ Department of Materials Science and Engineering National Cheng Kung University Tainan Taiwan; ^2^ Program on Semiconductor Packaging and Testing Academy of Innovative Semiconductor and Sustainable Manufacturing (AISSM) National Cheng Kung University Tainan Taiwan; ^3^ Program on Key Materials Academy of Innovative Semiconductor and Sustainable Manufacturing (AISSM) National Cheng Kung University Tainan Taiwan; ^4^ Taiwan Consortium of Emergent Crystalline Materials (TCECM) National Science and Technology Council Taipei Taiwan; ^5^ Department of Physics National Cheng Kung University Tainan Taiwan; ^6^ School of Electrical Engineering and Computer Science University of Ottawa Ottawa Canada

**Keywords:** GeSe_2_, light power, multi‐valued logic, photodetector, trap state

## Abstract

Germanium diselenide (GeSe_2_), a binary IV–VI chalcogenide material, holds immense potential for ultra‐violet (UV) optoelectronics due to its exceptional light‐absorption efficiency and chemical stability. Among the existing polymorphs, including α‐, β‐, and γ‐GeSe_2_, the β phase with layered features standing to the relatively thermodynamic stable configuration has been investigated, whereas the photodetection feasibility is limited within the UV spectral regions. We affirm the phase engineering to transform the single‐crystallite β‐GeSe_2_ bulk crystals into the air‐stable β/α GeSe_2‐x_ nanoparticles, where the formation of the α‐GeSe_2_ phase triggered by the solid‐state ball milling has never been experimentally revealed. From investigating the conduction mechanism, characterizing the photophysical origin, and uneviling the band features, the mediations of dual trap states are validated to access the β to α phase transformation and morphological change. The hybrid photodetector design, by means of incorporating PMMA coating with GeSe_2‐x_ nanoparticles, features the light‐power‐resolved characteristics and exceptional photodetection performance upon detecting the visible 525 nm light, with a flicker noise amplitude of 4.5 × 10^−12^ A Hz^−1/2^ and response time/decay time of 9.6/6.7 µs. We further showcase the photonic multi‐valued logic circuits triggered by the light‐power modulation, enabling the optical ternary NOT gate to perform the logic inversions for ternary levels.

## Graphical abstract

We report the solid‐state ball milling, a traditional, reliable, mass‐productive material processing, to prepare the air‐stable and dual‐phase GeSe_2‐x_ nanoparticles with extended photodetection feasibility toward optical‐wavelength regions. We further display photonic multi‐valued logic (MVL) circuit through the employment of a hybrid PMMA/GeSe_2‐x_ based photodetector.

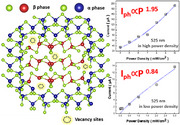

## 1 Introduction

Implementation of photodetector design, acting an indispensable role in diverse applications including military early warning, environmental monitoring, and wearable optoelectronic devices, has demanded ultra‐high sensitivity, low‐power consumption, and fast response. Conventional wide‐bandgap semiconductors, including AlGaN [1], Ga_2_O_3_ [2], SiC [3], and ZnO [4], are often constrained by costly and complex epitaxial processes as well as stringent lattice‐matching requirements, which hinder their practical employment and feature less compatibility with current microelectronic technology. Two‐dimensional (2D) materials, owing to their atomic‐scale thickness, spatially layered configuration, and tunable optoelectronic characteristics, exhibit excellent compatibility with silicon (Si)‐based electronics and can mitigate the performance degradation associated with lattice mismatch and interfacial defects in conventional crystalline semiconductor heterostructures. In addition, their high surface‐to‐volume ratio, strong light–matter interaction, and inherent flexibility enable their integration into flexible and wearable optoelectronic sensing platforms for intelligent human–machine interfaces [5, 6]. Among the various candidate 2D semiconductor materials, germanium diselenide (GeSe_2_) exhibits a layer‐independent wide direct bandgap of ∼3.0 eV [7], due to its remarkable absorption coefficient of ∼10^4^ cm^−1^ [8], excellent air stability, and sustainability in earth crust [9]. GeSe_2_, a typical binary IV–VI chalcogenide with a wide bandgap, crystallizes in three phases, including orthorhombic α‐GeSe_2_, monoclinic β‐GeSe_2_, and hexagonal γ‐GeSe_2_ [10]. Basically, the α phase, which forms at relatively low temperature [10], belongs to the orthorhombic lattice, where the lattice of GeSe_4_ tetrahedra is corner‐shared, forming a three‐dimensional (3D) network, which essentially displays a low density of crystal defects and prefund crystallite robustness [11, 12].

In contrast, β phase, a relatively thermodynamic stable lattice configuration with lower formation energy than that of other phases, is typically stabilized at elevated temperatures, adopting a monoclinic structure consisting of corner‐ and edge‐sharing GeSe_4_ tetrahedra arranged in a 2D framework [13]. Moreover, β phase preserves the weaker interlayer interactions and features a layered architecture, which facilitates the pronounced carrier conduction characteristics due to its high carrier mobility. However, for photodetector applications, its layered nature renders it more susceptible to uncontrollable structural disorder and interfacial defects [14], resulting in serve electric leakage and alleviated response speed. The distinct phase‐dependent features of GeSe_2_ provide considerable flexibility for device engineering, yet these issues have rarely addressed, and the existing studies primarily focused either on single β‐phase GeSe_2_ or external heterostructures [15, 16]. Meanwhile, recent studies on wafer‐scale 2D semimetals and van der Waals hybrid heterostructures, including PtTe_2_, PdTe_2_, MoTe_2_, WTe_2_, and NiTe_2_‐based systems, have demonstrated the broadband and mid‐infrared photodetection characteristics, highlighting the importance of scalable materials integration, low‐noise operation, and fast carrier extraction in advanced optoelectronic sensing platforms [17, 18, 19, 20, 21, 22]. Nevertheless, the strategy of heterostructure engineering suffers from the adverse effects from lattice mismatch and structural asymmetry that evolves the serves interfacial defects, restricting further improvements in photoresponses and detection dynamics. For example, Yang et al. demonstrated that β‐GeSe_2_ exhibits excellent polarization‐sensitive response in the short‐wavelength region. However, their devices solely rely on mechanically exfoliated flakes, where the device integration is size‐limited and difficult to scale up, and required operation at a relatively high bias (10 V), leading to excessive dark leakage and power consumption unsuitable for practical assessment [23]. Zhou et al. reported rhombic β‐GeSe_2_ flakes on mica substrates via chemical vapor deposition and achieved the anisotropic photoresponse depending on the polarization of detected light [8]. Yet, the unavoidable interfacial defects introduced during the transfer process significantly degrade the photoelectric conversion efficiency. Wu et al. reported wafer‐scale synthesis of wide‐bandgap 2D GeSe_2_ layers and demonstrated GeSe_2_/GaN van der Waals heterojunction photodetectors with self‐powered operation capabilities [24]. While their device structure successfully addresses the challenge of large‐area growth and achieved ultrahigh UV‐to‐visible rejection, the sole‐phase β‐GeSe_2_ still suffers from relatively large leakage interference and limited response speed. Moreover, so far, the present GeSe_2_‐based designs in the literature show the photodetection feasibility at UV spectral regions (mostly less than 360 nm in wavelength) [5, 7, 8, 15, 24], and noise analysis was rarely conducted. These unexplored aspects of device reliability and practical applicability remain to be highly demanded [15].

To overcome the fundamental bottlenecks of single‐phase systems and extrinsic heterostructure designs, the concept of phase heterojunction (PHJ) [25, 26, 27, 28, 29, 30, 31], which involves junctions between different phases of the same material, has recently attracted increasing attention. For instance, in the case of κ/β‐Ga_2_O_3_, the band offset between distinct polymorphs forms a type‐II staggered band alignment, establishing a strong built‐in electric field at the interfaces [25]. This induced field effectively promotes the separation of photogenerated carriers, achieving a responsivity as high as 17.8 mA W^−^^1^ at zero bias, which is three orders of magnitude higher than that of single‐phase devices, while the response and recovery times are reduced to 0.21/0.53 s. These results demonstrate that multiphase cooperation within the same material not only overcomes the intrinsic performance bottlenecks of single phases but also circumvents the challenges of lattice mismatch and interfacial defects inherent to traditional heterostructures, thereby combining both improved performance and process compatibility. Differences in phase within nominally the same material would be viable to modulate the physical and chemical properties, affecting the carrier mobility, chemical stability, energy bandgap, and more. Inspired by these findings, we report for the first time an α/β‐phase hybrid design in a single GeSe_2_ nanoparticle, where the interior 2D β‐phase frameworks envision the effective carrier condition, and the shell networks of 3D α‐phase constitutes sustain the crystallite robustness and provide the complementary band alignment and an internal electric field without relying on external heterostructure stacking. The present systematic investigations on the intrinsic heterostructures within a single material correlated with defect evolutions open up an unpresented while strategic routes enabling simultaneous improvements in demanding merits of photodetection performances upon power‐resolved responsivity, response dynamics at µs level and features of noise suppression.

## 2 Results and Discussion

### 2.1 Materials Characterizations

**FIGURE 1:**
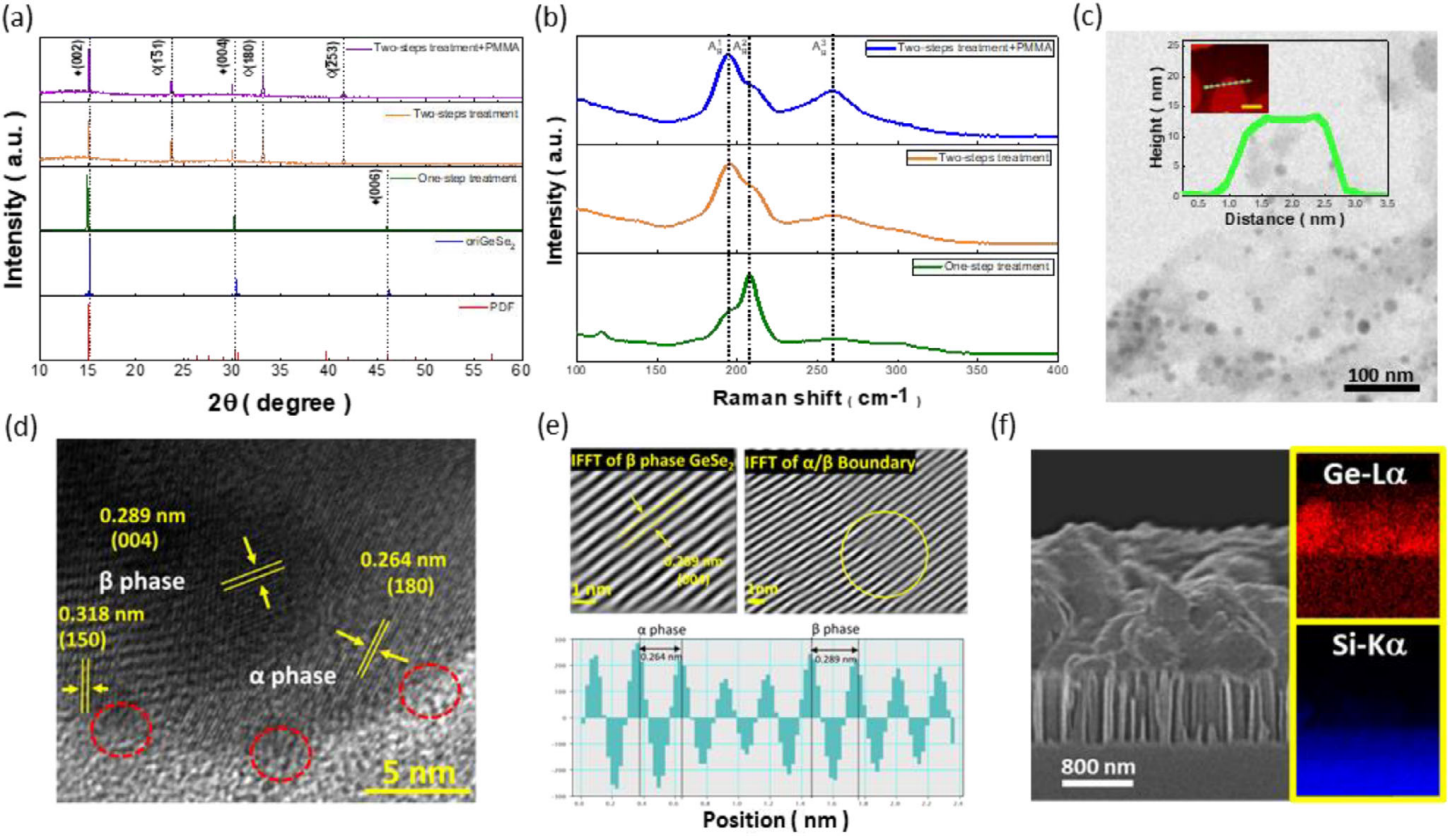
Materials characterizations of treated GeSe_2‐x_ nanoparticles: (a) XRD patterns of GeSe_2‐x_ samples subjected to different treatments.(b) Raman spectra of GeSe_2‐x_ samples under different processing conditions.(c) Morphology of GeSe_2‐x_ nanoparticles after ball milling (inset: thickness analysis). (d) HRTEM image of GeSe_2‐x_ with a dual‐phase structure. (e) Inverse fast Fourier transform (IFFT) results highlighting the β‐phase and α/β phase boundary in GeSe_2‐x_. (f) Cross‐sectional SEM image illustrating the PMMA/GeSe_2‐x_ layer on SiNR/Si substrates. EDS mappings of correlated heterostructures via probing characteristic X‐rays of Si‐Kα (figure above) and Ge‐Lα (figure below) are also presented.

Crystallographic investigations of two types of prepared GeSe_2‐x_ nanoparticles are performed with XRD characterizations, including one‐step and two‐step Se‐vacancy (V_Se_) engineered GeSe_2‐x_, as demonstrated in Figure 1a. The reference samples, name as pristine GeSe_2_ sheets, fabricated via the vertical gradient freeze method, are also displayed. The results reveal the consistent diffraction patterns of one‐step V_Se_ engineered GeSe_2‐x_ nanoparticles with pristine GeSe_2_, featuring the main characteristic reflections at (002), (004), and (006), as indexed in the JCPDS database (PDF#42‐1104). These findings disclose the retained layered nature and β‐phase configurations [9, 31]. The obtained GeSe_2‐x_ samples prepared with two‐step V_Se_ engineering, disclose the additional crystallographic peaks that emerge at (15̅1), (180), and (2̅53), which can be assigned to the α phase of GeSe_2_ [32]. The prolongation of the ball‐milling process essentially increases the relative intensity of crystallographic signatures of α phase, indicating that the imposed mechanical stress arising from mechanical compaction and intense collision between chemically‐stable ZrO_2_ balls and target GeSe_2_ nanoparticles refines the microstructures of GeSe_2_ lattices and drives a structural phase transition. Detailed XRD analyses of the obtained GeSe_2‐x_ fine nanoparticles under different milling durations are displayed in Figure S1. To probe the involved lattice crystallinity in β‐to‐α phase transformation of V_Se_ engineered GeSe_2‐x_, Raman characterizations are employed, as displayed in Figure 1b. It is noted that the α‐ and β‐phase vibrational features of GeSe_2_ are assigned to be approximately 200 and 210 cm^−^^1^, respectively, while the broad features around 267 and 305 cm^−^^1^ are associated with Se–Se pairs and amorphous Ge/Se components [24]. In the present Raman spectra, the crystallinity of pristine bulk GeSe_2_ sheets and mechanically exfoliated GeSe_2_ retain the pure β‐phase structures, identified by the pure characteristic signature located at 210 cm^−^^1^ [8, 33] (Figure S2). After undergoing the first‐step V_Se_ engineering treatment, the main β crystallite feature remains, whereas the slight broadening of full‐width half‐maximum (FWHM) is visualized (∼20 cm^−1^) compared with untreated and defect‐free β GeSe_2_ (< 11 cm^−1^), suggesting the formation of Se‐deficient GeSe_2_‐x lattices. Meanwhile, another characteristic signature is the appearance of addition Raman peak at 198 cm^−^^1^, which corresponds to the crystallographic configuration of α phase [8]. These results indicate the local structural changes associated with the mechanically induced re‐configuration of GeSe_2‐x_ via a sonication treatment. Subsequent ball milling as the second‐step treatment is found to explicitly signify the Raman signatures of α phase at 198 cm^−^^1^, which reveal the evident phase transformation of β‐to‐α and is consistent with the XRD results [34]. Apart from that, further employment of PMMA coating shows the trivial effect on the crystallite patterns and structural phases of GeSe_2‐x_ samples (Figure 1a,b), whereas the correlated impacts on noise suppression of photodetection characteristics will be discussed later.

In addition to the structural examinations, the quantitatively elemental ratio of Se and Ge from two types of engineered GeSe_2‐x_ is characterized by ICP‐MS analyses, indicating the atomic Se/Ge ratio of 1.98 and 1.61 via one‐step and two‐step V_Se_ engineering processes, respectively, as evidenced in Figure S3. Trace Zr signals originating from the ZrO_2_ milling media are detected only in the fully digested powder samples by ICP analysis, with the estimated contents of 0.2 and 0.9 wt.% from the one‐ and two‐step V_Se‐_engineered GeSe_2__−__x_ samples, respectively. During the subsequent solution process, the abundant Zr‐containing residues are readily precipitated and mostly excluded from the well‐dispersed GeSe_2__−__x_‐nanoparticle suspensions used for the film deposition. Consistently, no detectable Zr signal is observed in the EDS analysis or in the XPS survey spectrum of the hybrid PMMA/GeSe_2__−__x_ films. In addition, the V_Se_ concentration of samples after undergoing one‐step and two‐steps treatment is 4.0% and 20.5%, respectively. The non‐stoichiometric ratios of Ge to Se are further examined with quantitative EDS mappings (Figure S4) and integrated XPS signatures (Figure S5), showing the atomic ratio of Ge to Se 1.98 from EDS results (∼1:2 from XPS results) and 1:1.61 from EDS results (1:1.64 from XPS results) via one‐step and two‐steps V_Se_ engineering processes, respectively, which correspond to the results obtained from ICP‐OES investigations. The slightly higher Se contents (3%) of two‐steps V_Se_ treatment extracted from integrated XPS analysis relative to ICP‐MS and EDS quantitation can be interpreted by the fact that the escape depth of photoexcited electrons from GeSe_2‐x_ samples are spatially limited to less than 10 nm in depth, and thus the signal contributions from inner Se vacancies are hard to be detected. To further probe the chemical compositions and valence states of the obtained GeSe_2‐x_ via one‐step and two‐step processing treatment, high‐resolution XPS examinations are performed, as illustrated in Figures S5. Compared with sole Ge^4+^ signatures spectrally located at 1253.2 eV (2p_1/2_) and 1220.2 eV (2p_3/2_) obtained from the pristine GeSe_2_ crystals, the deconvoluted Ge 2p features can be classified with Ge^4+^ (2p_1/2_, 1252.5 eV and 2p_3/2_, 1220.1 eV) and Ge^2+^ (2p_1/2_, 1248.3 eV and 2p^3/2^, 1218.5 eV) in the cases of one‐step and two‐steps V_Se_ engineered GeSe_2‐x_. The present redshifts explicitly illustrate the loss of anion coordination within the bonding environments due to V_Se_ evolution, and further modulate the electronic structures. Furthermore, EPR spectra (Figure S6) of two types of prepared GeSe_2‐x_ disclose a noticeable signature at g = 2.003 [35] from one‐step V_Se_ engineering treatment, and the correlated feature is explicitly signified from two‐step V_Se_ engineering treatment, which reveals the increase of V_Se_ spatial density promoted by interfacial phase transformation and surface dangling configurations.

Morphologies of two‐step V_Se_ engineered GeSe_2‐x_ are visualized via the TEM characterizations, as displayed in Figure 1c. The nanoparticle features of obtained GeSe_2_‐x can be observed, displaying an average size of 12.9 nm with a standard deviation (SD) of 2.54 nm, estimated by the statistic determination of size distribution obtained from TEM observations, as indicated in Figures S7 and S8 (To ensure the fabrication reproducibility, the average sizes (SD) of samples fabricated in a second run and third run are 13.3 nm (2.78 nm) and 12.8 nm (3.05 nm), respectively). Given the monolayer thickness of GeSe_2_ lattices around 0.70 nm, each GeSe_2‐x_ nanoparticle is estimated to consist of 9–17 crystallite layers [24, 36]. Topographic AFM examination on scanning the lateral profiles of a single GeSe_2‐x_ nanoparticle visualizes the thickness of around 13.1 ± 0.4 nm. A representative HRTEM characterization is presented in Figure 1d, where two distinct lattice fringes are clearly labeled: (004) planes of β phase with an interlayer spacing of 0.289 nm at inner sites of a nanoparticle and (180) planes of α phase with interlayer spacing of 0.264 nm at the outer sites, identifying the coexistence of β/α phases at a single GeSe_2‐x_ nanoparticle. In addition, the partially irregulated features at a nanoparticle surface marked in red circle are attributed to the ball‐milled induced surface defects. Microscopic visualization can be further realized by monitoring the microstructure evolutions via Inverse Fast Fourier‐transform (IFFT) analysis, as demonstrated in Figure 1e. The correlated IFFT patterns of physically exfoliated GeSe_2_ sheets display pure β‐phase regulation without appearing the visible lattice boundary and crystal defects, as shown in the left scheme of Figure 1e. The long‐range crystallite ordering is evidenced from a two‐step engineered GeSe_2‐x_ nanoparticle when spatially monitoring the microstructures at the central and edge regions of a nanoparticle, confirming the treatment does not destroy the primary crystal structures. In addition, the explicit β/α phase boundary, labeled at the yellow circle in the right scheme of Figure 1e, is observed that separates two distinct crystallite lattices with unequal lattice constants and configurations.

During the solid‐state ball milling, the repeated mechanical collisions induce the localized strain and coordination disturbance within the β‐GeSe_2_ framework [37, 38]. It has been reported that high‐energy ball milling can thermodynamically decrease the crystallization temperature and activation energy of the residual amorphous phase, indicating that mechanical deformation can promote the phase transformation [39]. In the present GeSe_2__−__x_ system, the partial Se loss and V_Se_ formation can destabilize the original layered β‐GeSe_2_ coordination environment and provide the additional configurational freedom for Ge–Se bond rearrangement. Such vacancy‐mediated bond rearrangement allows local stress relaxation, especially near nanoparticle surfaces and β/α interfacial regions where the coordination constraint is weaker than that in the bulk lattice. Therefore, the observed α‐phase component is more appropriately interpreted as a mechanically assisted and V_Se_‐mediated local structural reconstruction rather than a complete bulk β‐to‐α conversion [39]. The retained crystalline features in XRD patterns, the β/α lattice fringes in HRTEM/IFFT, and the indexed SAED rings/spots further indicate that the ball‐milled nanoparticles preserve the ordered β/α crystalline domains with local surface disorders, rather than undergoing the complete mechanochemical vitrification. Additional SAED analysis and semi‐quantitative Raman peak‐deconvolution results are provided in Figures S9 and S10 and Table S1, further supporting the coexistence of β‐ and α‐phase domains in the ball‐milled GeSe_2__−__x_ nanoparticles.

### 2.2 Conduction Mechanism of Vacancy‐Induced GeSe_2_

**FIGURE 2:**
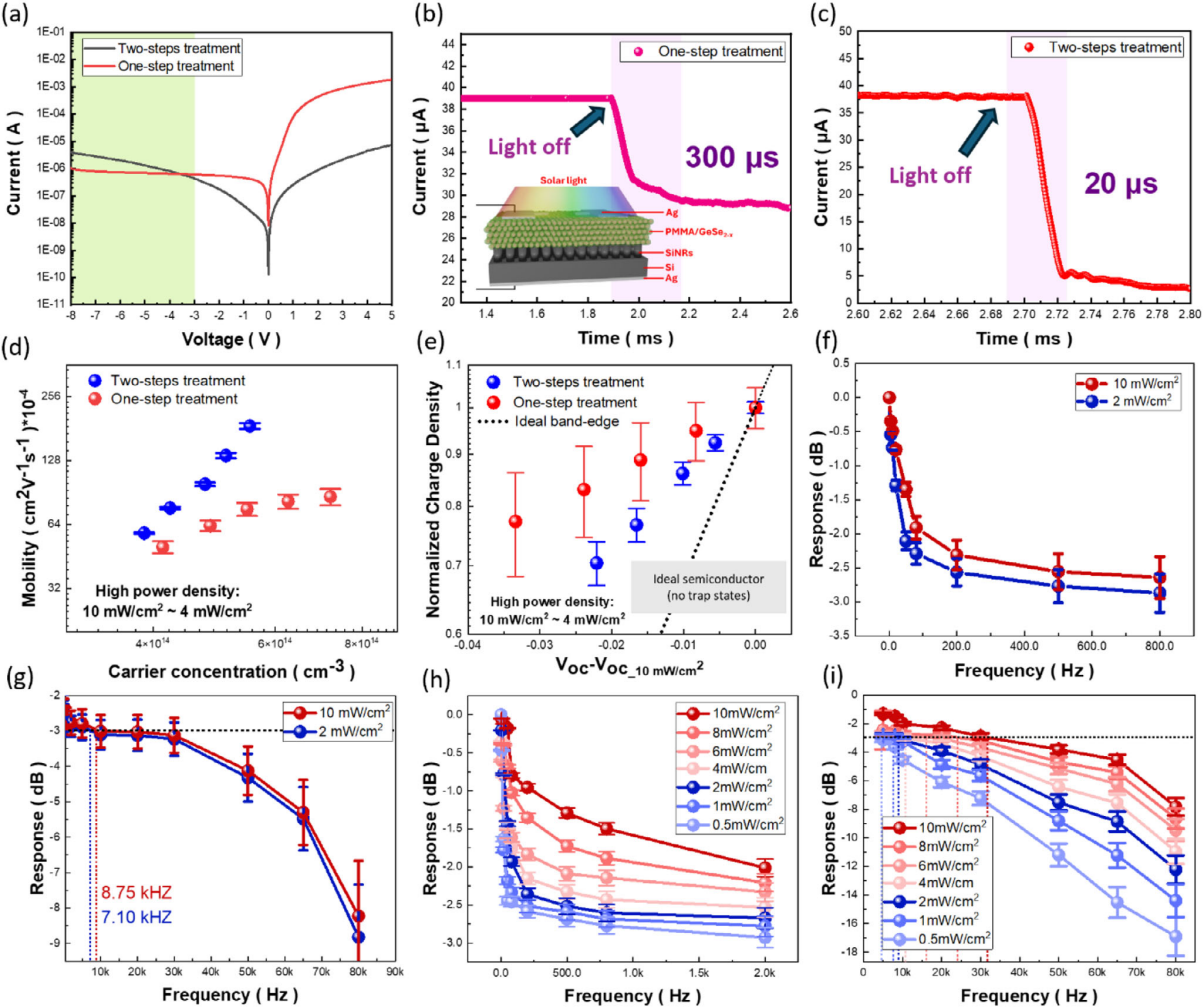
Investigations of correlated conduction mechanism: (a) Dark *I*–*V* curves in a semi‐log plot. Light on/off transient photocurrent decay with the light intensity of 10 mW/cm^2^ illumination at ‐1 V from (b) one‐step V_se_ and (c) two‐step V_se_ treated GeSe_2‐x_. In addition, the schematic illustration of vertical *I*–*V* measurements under the irradiations of simulated solar lights is presented in the inset of Figure 2b. (d) Effective charge carrier mobilities as a function of charge carrier densities were analyzed by charge extraction at 1 V at the light intensity range of 10^−4^ mW/cm^2^. (Average SD values: Two‐steps ± 2.02% and one‐step ± 8.74%). (e) Normalized plots of the charge carrier density at open circuit versus open circuit voltage determined from integration of charge extraction transients as a function of light irradiation intensity (data normalized at 10 mW/cm^2^ of light intensity) (Average SD values: Two‐steps ± 2.88%, one‐step ± 8.36%). −3 dB cutoff frequency responses of GeSe_2‐x_ (one‐step V_Se_ engineering) in both low‐ (Figure 2f) and high‐frequency (Figure 2g) regimes. For Figure 2f, the average SD values are ± 8.27% (10 mW/cm^2^) and ±6.62% (2 mW/cm^2^). Figure 2g, the average SD values are ± 15.33% (10 mW/cm^2^) and ± 13.72% (2 mW/cm^2^). −3 dB cutoff frequency responses of GeSe_2‐x_ (two‐steps V_Se_ engineering) in both low‐ (Figure 2h) (Average SD: ± 2.78%, ± 2.91%, ± 3.10%, ± 3.38%, ± 3.68%, ± 3.88%, and ± 4.32% for 0.5–10 mW/cm^2^) and high‐frequency (Figure 2i) (Average SD: ±6.38%, ±6.64%, ±6.78%, ±6.84%, ±7.03%, ±6.91%, and ±6.81% for 0.5–10 mW/cm^2^) regimes.

Optoelectronic correlations between trap states and the photoresponsive output of GeSe_2_‐x photodetectors are undertaken via a series of conduction characteristics under dark and AM 1.5 G solar illuminations, respectively. Cross‐sectional SEM observation displays the overall active structures of hybrid photodetectors (Figure 1f), comprising of a layer of PMMA@GeSe_2_‐x nanoparticles (layer thickness = 1.6 ± 0.09 µm) via a solution‐processed deposition on SiNR/Si substrates. In addition, the results of EDS mappings probing Ge‐Lα (figure above) and Si‐Kα (figure below) support the successful fabrication of devices. Essentially, the hybrid device consists of a SiNR/Si as the substrate that provides structural support and suppresses the undesired leakage currents from Si (Figure S9), coated with a PMMA/GeSe_2_‐x hybrid layer serving as the primary light absorber and carrier‐transport medium, and a pair of Ag electrodes on the device top as electric outputs for photodetection operation. To unveil their optoelectronic origins and conduction physics, optoelectronic and electric measurements are conducted in vertical configuration under the irradiations of simulated solar lights: Ag (top electrode)/PMMA@GeSe_2_‐x/SiNR/Si (rear electrode) (inset of Figure 2b), where the systematic examinations are presented in Figure 2. First, Figure 2a reveals that the reverse saturation currents at dark under the sweeping external bias from +5 to ‐8 V. The dark currents at negative external potentials of one‐step V_Se_ engineered GeSe_2‐x_ samples closely achieve the saturation at sweeping regions of 0 to ‐3.5 V, whereas the obtained reverse dark currents from the sample treated with two‐step V_Se_ engineering remain unsaturated at input bias across ‐3.5 V and extend to the offset voltage of ‐8 V. The results elaborate the presence of deep trap states within two‐steps V_Se_ engineered GeSe_2‐x_ that modulate the conventional band‐like photoresponses of semiconductor‐type 2D materials into defect‐mediated power‐dependent photodetection responses. As visualized in the microstructural characterizations shown in Figure 1, the GeSe_2‐x_ nanoparticles obtained from the two‐step V_Se_ engineering process contain the Se‐vacancy configurations at both lattice and surface/interface regions. The lattice‐related V_Se_ configurations are associated with shallow localized states near the band‐edge region, whereas the surface/interface‐related V_Se_ configurations introduced during the ball‐milling process contribute to the deeper localized states. These spatially distinct defect configurations provide the structural basis for the dual‐trap‐mediated carrier dynamics discussed below. The involvement of surface/interface‐related deep traps is also correlated with the ball‐milling process, as supported by the dark *I*–*V* characteristics of samples prepared with different milling durations in Figure S11 [40, 41]. These results indicate that the deep‐trap contribution affects the carrier extraction under the low electric fields and carrier release under the stronger reverse bias.

To further clarify the conduction model, the *I*–*V* characteristics are replotted in a log(I)–log(V) format under the dark and illuminated conditions with different light intensities, as shown in Figure S12 and summarized in Table S2. Under the dark and low‐power illumination conditions, the results exhibit the SCLC‐like trap‐limited conduction behavior, including an ohmic region with a slope close to unity and a trap‐filling region with a slope larger than 2. This behavior indicates that the carrier transport is strongly affected by the vacancy‐induced trap states under the weak excitations. In contrast, under the high‐power illuminations, the log(I)–log(V) profiles deviate from the conventional voltage‐controlled SCLC trend, suggesting that the trap states are progressively filled by the photogenerated carriers and that the transport behavior turns to be dominated by the light‐assisted carrier extraction and drift transport. Therefore, the conduction mechanism evolves from trap‐limited SCLC‐like transport at the low illumination conditions to trap‐filled, photoconductive drift‐assisted transport at the high illumination conditions.

Examinations on charge extraction (CE) and transient photovoltage of are employed to probe the shallow‐trap‐mediated carrier transport under a pulse‐light illumination with a high power density (10 mW/cm^2^) [42], as displayed in one‐step V_Se_ engineered (Figure 2b) and two‐step V_Se_ engineered (Figure 2c) GeSe_2‐x_, respectively. The transient photocurrent decay of one‐step V_Se_ engineered GeSe_2‐x_ features a relatively large decay time up to 300 µs. This can be understood by the fact that the involvement of shallow traps in GeSe_2‐x_, induced by V_Se, lattice_, prolongs the average residence time of carriers near the band edge through the repeated capture–release cycling of carrier dynamics. The results can be further supported by the slower photoresponse delays (within 50 to 300 µs) of samples when elongating the sonication duration up to 6 h, which manifests the evident correlation between structural V_Se, lattice_ evolution, and transient delay of current recovery, as demonstrated in Figure S13.

On the other hand, the GeSe_2‐x_ sample treated with two‐step V_Se_ engineering procedures, envisions the explicitly rapid photocurrent decay (20 µs), approximately 15 times faster than that of the sonication‐only process (∼300 µs), as demonstrated in Figure 2c. The results address the much faster response dynamics relative to the transient PL decays, featuring the explicitly rapid charge extraction overwhelming the radiative limit [43], which can not be solely interpreted by the defect‐induced recombination [42, 43], yet these reflect the involvement of competing trapping mechanisms owing from spatial and spectral differences of trap distributions. The charge trapping/detrapping features are alleviated because the relatively low shallow‐trap density existed in two‐steps V_Se_ engineered GeSe_2‐x_, which intends to release mobile carriers due to the larger drift field in reverse bias once the deep traps are filled (Figure 2a), contributing to the accelerate charge extraction. Thus, we conclude that the transient origin of two‐steps V_Se_ engineered GeSe_2‐x_ structure is dominated from the coexistence of shallow and deep traps, which suppresses the extent of carrier capture–release cycling dominated by the sole configuration of shallow trap states, thereby minimizing delayed recovery and producing faster photocurrent decay dynamics (Figure 2c). It should be noted that in Figure 2d, the difference of modulated drift mobilities using a drift‐transport model is observed from two sets of GeSe_2‐x_ samples by monitoring the short‐circuit carrier densities (J_sc_) excited at various light intensities, ranging from 4–10 mW/cm^2^, respectively [44]. In the case of one‐step V_Se_ engineered GeSe_2‐x_, the present mobility is greatly limited by the high shallow‐trap density and approaches to saturation when J_sc_ is above 6 × 10^14^ cm^−3^, aligned with the typical semiconductor 2D‐material based photodetectors, where the involved carrier‐conduction kinetics is dominated by the barrier heights of shallow traps [45]. However, the contradicted result is found from GeSe_2‐x_ structures engineered by two‐steps V_Se_ process, where the unsaturated mobility trend correlated with the leap of J_sc_ is visualized. The higher drifted carrier mobility is associated with the lower relative amount of shallow‐trap density existed in GeSe_2‐x_ due to the additional participation of V_Se, surface_‐induced deep trap states, which facilitates the carrier extraction and conduction characteristics under a light‐power photoexcitation. In this work, the shallow and deep trap states are distinguished according to their energetic positions, structural origins, and carrier‐dynamic responses. The shallow trap states are defined as vacancy‐induced localized states located close to the band‐edge region and are mainly associated with lattice V_Se_ configurations. These states participate in repeated carrier capture–release processes and therefore prolong the photocurrent recovery. This behavior is supported by the one‐step V_Se_ engineered GeSe_2‐x_ sample, which exhibits a relatively slow photocurrent decay time of approximately 300 µs. In contrast, the deep trap states are energetically located farther from the band edge and are mainly related to surface/interface V_Se_ configurations generated during the ball‐milling process. The two‐step V_Se_ engineered GeSe_2‐x_ sample exhibits a much faster photocurrent decay time of approximately 20 µs, suggesting that the additional involvement of deep traps modifies the carrier‐extraction pathway and suppresses delayed recovery once the traps are filled under sufficient illumination or reverse electric field. Moreover, the decay time is reduced from approximately 60 µs for bare GeSe_2‐x_ to approximately 20 µs after PMMA encapsulation, indicating that PMMA mitigates surface‐trap‐related recovery delay and improves carrier extraction.

To further examine the energetic distribution of shallow‐trap states, we analyze the dependence of open‐circuit voltage (V_OC_) on the modulation of carrier density, as presented in Figure 2e. In an ideal and trap‐free semiconductor, the response of carrier density illustrates the exponential increase with V_OC_, featuring the dashed line shown in Figure 2e. Nevertheless, the reduced illumination intensity lowers the quantity of V_OC,_ which in turn fosters the positive dependence to the light intensity that reflects a smaller quasi‐Fermi level splitting. Such feature associates with the slow reduction of extracted carrier density and deviates from the ideal correlation. The experimental slopes of both GeSe_2‐x_ samples obtained by one‐step and two‐steps V_Se_ engineering are notably smaller than the result predicted by the ideal model, indicating the presence of band‐tail states due to the evolution of shallow‐trap levels. Furthermore, the deviation is relatively significant in one‐step V_Se_ engineered samples, manifesting a relatively large energy spread of shallow traps near the band edge compared with a sample treated with two‐step V_Se_ engineering. These shallow traps act as temporary reservoirs of photogenerated charges, slowing the growth of carrier concentration triggered under the photoexcitations, and thereby reducing both linearity and stability of the photoresponse [46]. This trap‐limited extraction mechanism weakens the dependence of output photocurrents on the modulation of light‐illumination intensity, which thus degrades the feasibility of weak‐light detection. By contrast, the two‐step V_Se_ engineered sample exhibits a narrower band‐tail distribution and relatively lower shallow‐trap density, enabling the conduction behavior closer to the ideal drift transport and significantly modulating V_OC_ deviation, which further fosters the faster‐response characteristic at high light intensities. In addition, the characteristics of photocurrent decay behavior (60 µs) of GeSe_2‐x_‐based photodetectors in the absence of PMMA passivation is examined, as shown in Figure S14. We find that the hybrids containing two‐step V_Se_ engineered GeSe_2‐x_ and PMMA blends envision the suppressed recovery delays of chopped photoresponses, where accelerated switching behavior is found to be particularly dominant at low‐frequency circumstances (< 2000 Hz). The effects of polarization hysteresis from PMMA coating associate with the dipole evolution in PMMA chains that undergoes the orientational polarization at low‐frequency domain, whereas at high frequencies, the dipoles cannot follow the rapid field oscillations, leading to reduced polarization contributions [47]. These explain the positive contribution of blended PMMA coating on broadening the frequency bandwidth of photoresponsive GeSe_2‐x_, which improves the feasibility of photodetector operation. Additional UV/visible‐light absorption and operational stability measurements are provided in Figure S15 and S16. It can be found that the light absorption of a PMMA at the light wavelength of 525 nm is negligible, indicating that the improved photoresponse is not mainly caused by optical absorption enhancement. In contrast, the stability comparison confirms that PMMA effectively suppresses photocurrent degradation upon the operation, supporting its role as a surface/interface passivation and encapsulation layer.

Examinations on frequency characteristics with respect to low (2 mW/cm^2^) and high (10 mW/cm^2^) light intensities are performed on the GeSe_2‐x_ samples treated with one‐step V_Se_ engineering, where the corresponding frequency‐dependent photoresponses at low‐frequency and high‐frequency circumstances are depicted in Figure 2f,g, respectively. At the low‐frequency region (Figure 2f), the transient time of carrier capture/release are shorter because the shallow‐traps dominate the frequency‐dependent conduction origin, and the carriers reach the quasi‐equilibrium rapidly, rendering the cutoff frequency less sensitive to light intensity. At the high‐frequency region (Figure 2g), such an effect of rapid carrier capture/release is alleviated because of relatively faster alternating field polarizations, where the ‐3 dB cutoff frequency is visualized to be 7.10 and 8.75 kHz under the light intensities of 2 and 10 mW/cm^2^, respectively [48]. By comparing the low‐frequency results shown in Figure 2f [one‐step V_Se_ engineering] and 2 h [two‐steps V_Se_ engineering], one can clearly observe the explicit retardation of photocurrent decay relative to operation frequency (Figure 2h) in the case of the GeSe_2‐x_ sample treated with two‐step V_Se_ engineering. These findings can be attributed to the involvement of V_Se,surface_‐induced deep traps that decelerate the transient process of carrier transport. When increasing the levels of light input, the deep traps are gradually filled, and the evolution of the drift field due to charge accumulation drives the carrier conduction, and thus the present frequency‐dependent behaviors show the pronounced intensity‐dependent features, as evidenced in Figure 2h. At the high‐frequency region (Figure 2i), the capture efficiency of shallow traps is significantly reduced due to the rapid polarization‐alternating environment, which causes the shift of carrier‐transport origin to drift‐dominated conduction. Consequently, the resulting frequency response at high light‐intensity levels is kinetically determined by carrier extraction. As indicated in Figure 2d,e, the relatively rapid carrier‐extraction kinetics of deep‐trap assisted GeSe_2‐x_ are validated, and these support the ‐3 dB cutoff frequency reaching 31.74 kHz, beyond three times higher than that of one‐step engineered GeSe_2‐x_ (8.75 KHz) at high‐frequency circumstances. The detailed results of ‐3 dB cutoff frequency with respect to input light intensity are displayed in Table S3. Diving into the frequency‐monitoring responses in Figure 2f–i, the beneficial role of two‐step V_Se_ engineering treatments on preparing GeSe_2‐x_ is elaborated, where the dual traps are jointly dedicated to modulate the frequency response and raise the carrier transport speed at wide operation frequencies. Overall, the electrical and transient results indicate that the carrier dynamics cannot be explained by a single trap population. The one‐step VSe‐engineered GeSe_2__−__x_ is mainly governed by V_Se, lattice_‐induced shallow traps, whereas the two‐step ball‐milled GeSe_2__−__x_ additionally involves V_Se, surface_‐induced deep traps. The coexistence of these two trap populations accounts for the intensity‐dependent frequency response and accelerated carrier extraction under sufficient illumination or reverse electric field.

### 2.3 Photophysical Characterizations

**FIGURE 3:**
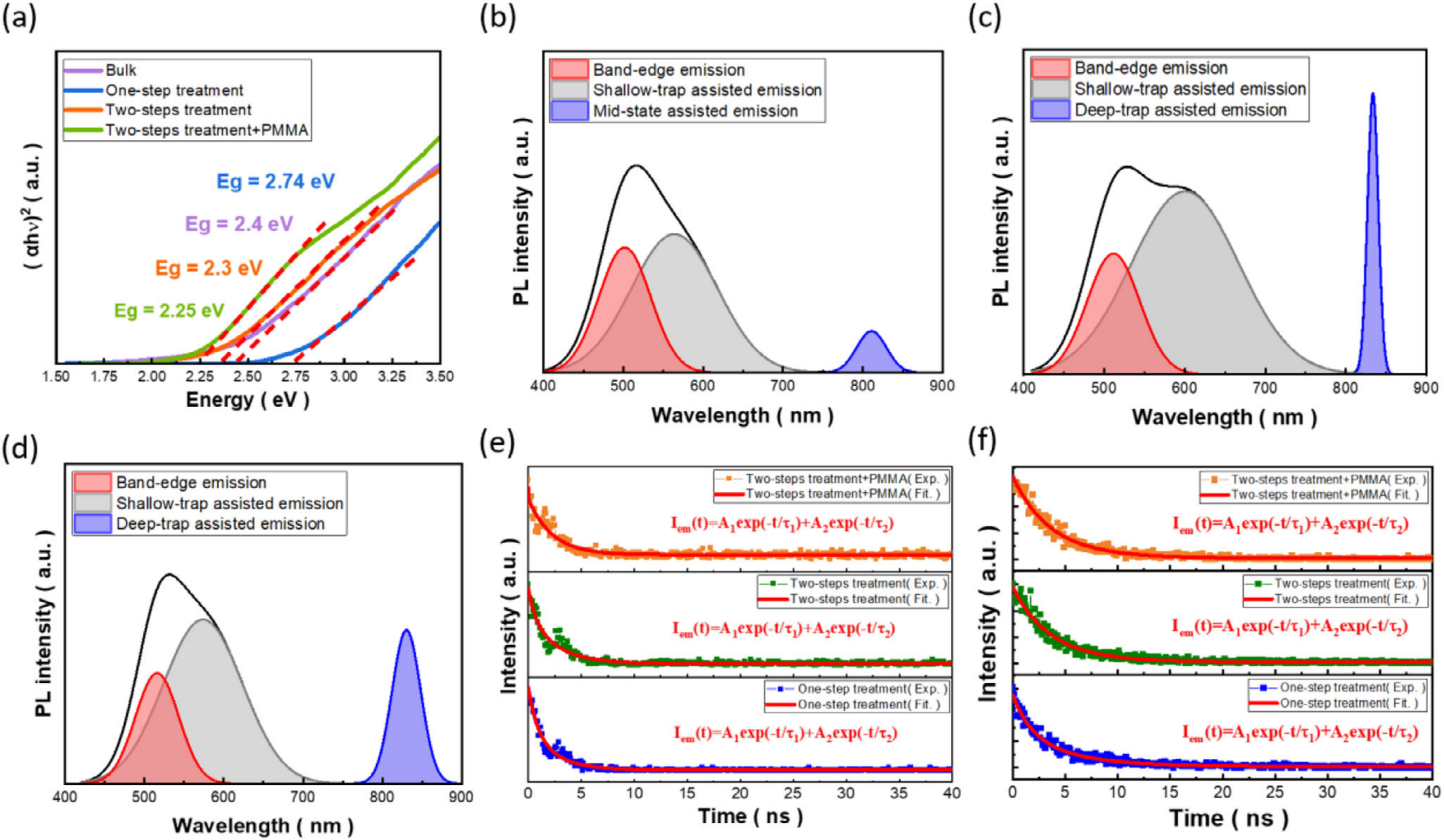
Photophysical characterizations of V_Se_ engineered GeSe_2‐x_: (a) Extracted bandgap energies of various GeSe_2_ samples. Static PL characteristics of (b) one‐step V_Se_ engineered GeSe_2‐x_ nanoparticles, (c) two‐step V_Se_ engineered GeSe_2‐x_ nanoparticles, and (d) hybrid PMMA/GeSe_2‐x_ obtained from two‐step V_Se_ treatment. Spectroscopic time‐resolved PL decay profiles of one‐step V_Se_ engineered GeSe_2‐x_, two‐step V_Se_ engineered GeSe_2‐x_, and hybrid PMMA/GeSe_2‐x_ monitored at (e) 800 nm and (f) 430 nm, respectively.

Figure 3a indicates the bandgap energies (E_g_) of GeSe_2‐x_ samples obtained from various treatments, where the displayed Tauc plots are readily extracted from the correlated UV–vis light absorption measurements (Figure S17), respectively. Compared with the measured bandgap energy of bulk and stoichiometric GeSe_2_ (E_g_ = 2.4 eV), the synthesized one‐step V_Se_ treated GeSe_2‐x_ sheets show relatively larger Tauc slopes, with estimated E_g_ of 2.74 eV from 6.0 h sonication duration, where the correlated results of GeSe_2‐x_ samples treated with various sonication durations are presented in Figure S18. These broadened spectral E_g_ offset reveal that the layer numbers of treated GeSe_2‐x_ are reduced to around six due to a significant quantum confinement [24, 36, 49]. The explicit decrease of Eg value (E_g_ = 2.3 eV) in the case of two‐step V_Se_ treatment is validated. This reveals that the additional ball‐milling procedure on nanoparticle formation not only causes the re‐arrangement of few‐layer GeSe_2‐x_ sheets being stacked into the flake features forced by the mechanical compression, but further induces the microscopic structural transformation into the formed α/β phases [7, 50, 51]. These combined effects explain the drop of E_g_ down to 2.3 eV from two‐steps V_Se_ treated GeSe_2‐x_ nanoparticles [16, 29, 40]. In addition, the incorporation of thin PMMA coating with the deposited GeSe_2‐x_ nanoparticle cause the slight reduction of measured E_g_ to 2.25 eV, originating from the additional interfacial states introduced at PMMA/GeSe_2‐x_ interfaces [52]. The results of lowering E_g_ account for the photodetection characteristics extending to visible wavelength regions, which will be investigated in the next section.

The strong correlation of spectral positions of defect states in crystalline GeSe_2‐x_ nanoparticles with luminescent characteristics is envisioned via the static PL analysis, as presented in Figure 3b–d. The primary PL signatures arising from band‐to‐band transition (γ_band_) can be found, where the redshift of γ_band_ of 514 nm (two‐step treatment) (Figure 3c) with respect to γ_band_ of 502 nm (one‐step treatment) (Figure 3b) is attributed to the combined effect of band tail extension, spectrally smearing the valance band maximum, and α‐phase formation. In addition, the displayed PL spectra are accompanied with the variations of PL phenomena due to defect‐state evolution: The appearance of PL peaks spectrally far from γ_band_ correlates with deep‐trap assisted emission, which are located at 832 nm (two‐step treatment) (Figure 3c) and 811 nm (one‐step treatment) (Figure 3b), respectively [53]. The additional PL peaks spectrally close to γ_band_ are attributed to shallow‐trap assisted emission and are located at 601 nm (two‐step treatment) (Figure 3c) and 564 nm (one‐step treatment) (Figure 3b), respectively. Critically, the explicit transition of deep‐trap assisted emission from a weak PL signal at 811 nm (Figure 3b) to a sharp, intense feature at 832 nm (Figure 3c) reveals the proliferation of deep‐defect density. With a PMMA encapsulation, the consistent trend of spectral redshift in γ_band_ around 3 nm and PL characteristics of deep defect states around 2–5 nm when introducing the PMMA coverage, supported by a drop of E_g_ and the partial annihilation of defect‐bound PL effects, respectively, as evidenced in Figure 3d.

To explore the dynamic photophysical phenomena of PMMA‐blended GeSe_2‐x_, time‐resolved spectroscopic PL study is conducted. By monitoring the photophysical dynamics at 800 nm, the correlated decay trace from three different GeSe_2‐x_ samples are well fitted using a double exponential function: I(t) = A_1_exp(−t/τ_1_)]+A_2_[exp(−t/τ_2_)], where the deconvoluted dynamic parameters (A_1_, τ_1_) and (A_2_, τ_2_) are originated from a rapid nonradiative recombination and slow radiative recombination in the presence of shallow trap states and deep trap states in GeSe_2‐x_ crystals, respectively, as indicated in Figure 3e and summarized in Table 1. In the two‐steps V_Se_ engineered GeSe_2‐x_, one can observe that the charge‐carrier dynamics favor to be govern by the slow decay channel, evidenced by the increase of weighted parameter, A_2_ (64.57% and 53.40% from two‐steps and one‐step V_Se_ engineered GeSe_2‐x_, respectively), Meanwhile, the substantial decrease in weighted ratio of A_1_/A_2_ (0.56 and 0.83 from two‐steps and one‐step V_Se_ engineered GeSe_2‐x_, respectively) is attributed to the effect of deep‐trap evolution under a ball‐milling treatment. This leads to the consequence of a slow radiative‐recombination pathway as the dominant channel that triggers the relatively longer average carrier lifetime (τ_avg_ = 2.33 ns) with respect to the case of one‐step treated GeSe_2‐x_ (τ_avg_ = 2.01 ns). The further visualization of τ_avg_ can be elaborated with the deconvoluted carrier lifetimes in viewing the decreased nonradiative channel (τ_1_ = 0.44 and 0.92 ns from two‐step and one‐step V_Se_ engineered GeSe_2‐x_, respectively). These suggest that the trapped electrons in shallow trap states are energetically plausible to transfer to the deep trap states or directly excite to the unoccupied states above the Fermi level, attaining the results of A_1_/A_2_ reduction and a leap of τ_avg_. By introducing a PMMA coating, the reduced density of shallow traps due to the PMMA passivation effectively increases the weighted ratio of A_1_/A_2_ down to 0.20, contributed by the signified detrapping effect [54], which maintains the dominance of the radiative recombination channel supported by deep trap states.

**TABLE 1:** Extracted parameters from time‐resolved spectroscopic PL study.

430 nm	A_1_	τ_1_	A_2_	τ_2_	A_1_/A_2_	τ_avg_
One‐step	47.268	1.78	42.14	5.35	1.12	4.38
Two‐steps	53.15	3.04	40.67	4.57	1.30	3.86
Two‐steps +PMMA	62.52	3.10	39.96	4.85	1.56	3.98
800 nm	A_1_	τ_1_	A_2_	τ_2_	A_1_/A_2_	τ_avg_
One‐step	49.14	0.83	53.40	2.39	0.92	2.01
Two‐steps	28.85	0.56	64.57	2.50	0.44	2.33
Two‐steps +PMMA	13.99	0.002	69.89	2.26	0.20	2.26

We further probed the photoexcitation dynamics at 430 nm to clarify the band‐edge and shallow‐trap‐assisted relaxation behavior, as presented in Figure 3f and summarized in Table 1. Compared with the 800 nm decay, the 430 nm decay is more sensitive to near‐band‐edge relaxation and shallow‐trap‐assisted nonradiative processes. The decay‐time parameters, τ_1_ and τ_2_, are extracted from fitting the PL decay traces under a double exponential function, representing the carrier lifetimes of trap‐assisted nonradiative channel and band‐to‐band radiative channel, respectively [55, 56]. We elaborate that the explicitly raising A_1_/A_2_ ratios from 1.12 (one‐step treatment) to 1.30 (two‐step treatment) are mainly attributed to the generation of more electron traps via the additional ball‐milling procedure that favorably pumps to the rapid nonradiative channel. These cause the substantial reduction of τ_2_ (4.57 ns) and τ_avg_ (3.86 ns) in two‐step treated GeSe_2‐x_, with respect to those of one‐step treated GeSe_2‐x_ (τ_2_ = 5.35 ns and τ_avg_ = 4.38 ns). Incorporation of PMMA blends with two‐step GeSe_2‐x_ samples dramatically modifies the photophysical condition because the PMMA terminals essentially consist of electron‐withdrawing sites at oxygen‐containing groups and electron‐donating sites at methyl groups, which causes the polarization hysteresis under low polarization frequency (normally less than 2000 Hz) of illuminated lights [47] and in turn induces the transient dipole fields. Such involved dipolar circumstance localizes the electrons at surface traps of V_Se_‐engineered GeSe_2‐x_ that in‐turn effectively alleviates the carrier recombination nonradiatively and radiatively, displaying the increased τ_1_ (3.10 ns) and τ_2_ (4.85 ns), respectively. The consequence of dynamic delay in both channels gives rise to the increase of overall τ_avg_ to 3.98 ns. To further dive into the origin of suppression in carrier recombination, the correlated band structures of synthesized GeSe_2‐x_ (two‐step treatment) and hybrid PMMA/GeSe_2‐x_ are experimentally characterized with spectral analyses, as discussed in Figure S19. The slight reduction of work function (ϕ) in the cases of hybrid PMMA/GeSe_2‐x_ (ϕ = 4.32 eV) relative to bare GeSe_2‐x_ (ϕ = 5.16 eV) is visualized. Such electronic modulation can be attributed to the strong coupling of PMMA‐induced interfacial dipoles with GeSe_2‐x_ nanoparticles, which attracts the valence electrons, shifting to the vicinity of PMMA sides, causing an upper shift of the Fermi level. This drives more photoexcited electrons being trapped at deep‐trap levels spatially located at GeSe_2‐x_ surfaces, favorably boosting the carrier separation and increasing the carrier lifetime (Figure 3e). Overall, the emission features close to the band‐edge transition are associated with shallow‐trap‐assisted recombination, whereas the long‐wavelength emission is attributed to deep‐trap‐assisted states. The double‐exponential TRPL fitting further reveals the competition between fast trap‐assisted nonradiative decay and slower radiative recombination channels, confirming that the carrier dynamics are regulated by both shallow and deep trap states.

### 2.4 Photodetection Performances

**FIGURE 4:**
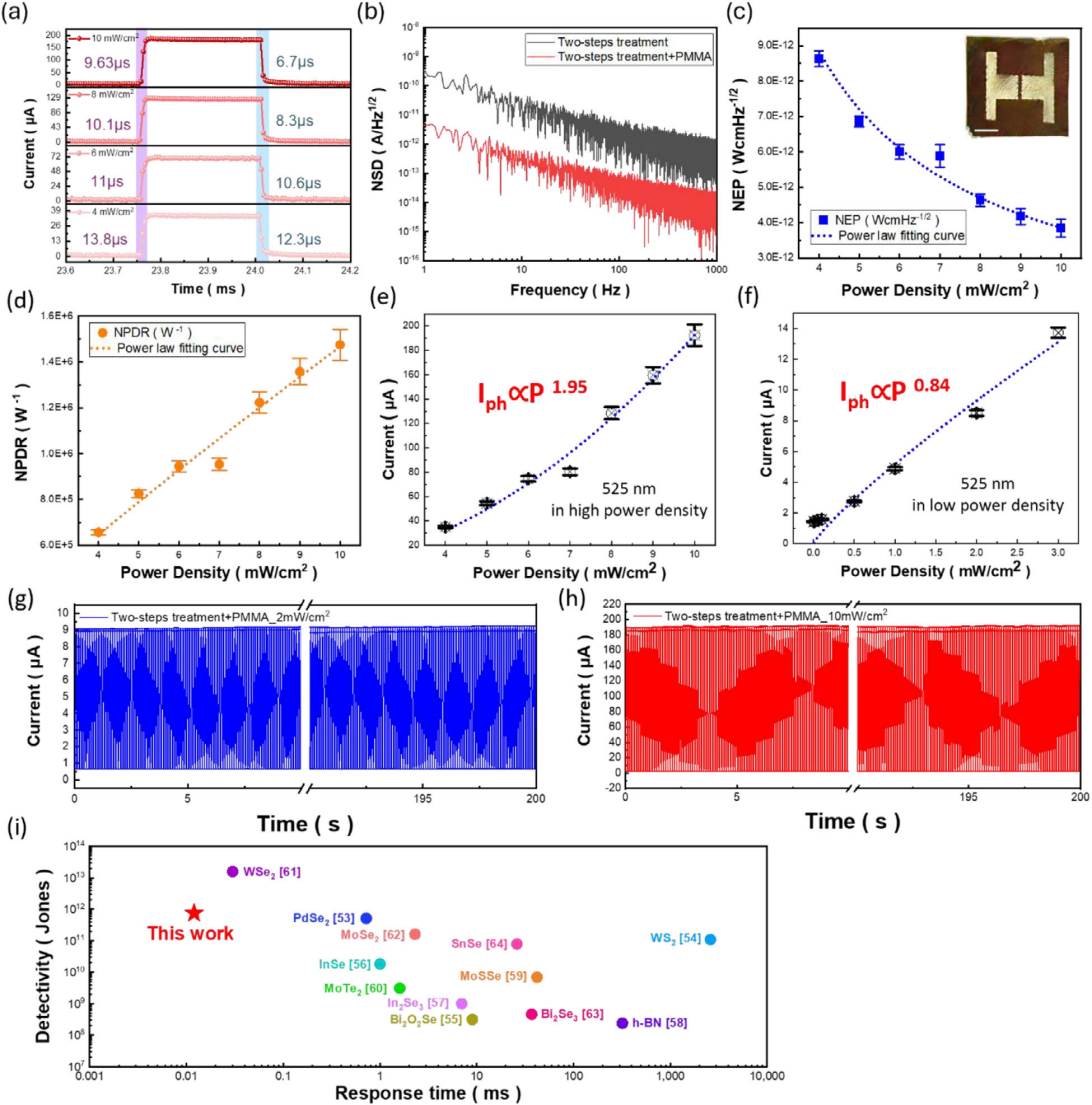
Evaluations of photodetection characteristics: (a) Temporal photoresponses and recovery dynamics of hybrid PMMA/GeSe_2‐x_ based photodetectors under various illuminated light intensities from 4–10 mW/cm^2^, respectively. (b) Frequency‐dependent NSD characterizations of GeSe_2‐x_ (two‐step treatment) and hybrid PMMA/GeSe_2‐x_ (two‐step treatment) based photodetectors. (c) NEP and (d) NPDR results of hybrid PMMA/GeSe_2‐x_ based photodetectors under various intensities of irradiated 532 nm lights. The inset figure displays the top‐view microscopic photograph of a fabricated device, where two Ag pads deposited on the PMMA/GeSe_2‐x_ layer are visualized (scale bar = 0.3 cm). The average SD values are ± 2.82% for Figure 4c and ± 3.10% for Figure 4d. In addition, the schematic illustration of a photodetector is presented in the inset of Figure 4c. Power‐resolved photoresponses under (e) high light power density (4–10 mW/cm^2^) and (f) low light power density (0.001–3 mW/cm^2^). The average SD values are ± 3.47% for Figure 4e and ± 1.60% for Figure 4f. The correlated features of the long‐term on/off switch under 1000 cycles are visualized with (g) high power density and (h) low power density of light irradiations, respectively. For Figure 4g, the average SD values in the initial 0–10 s region are ± 2.21% (light‐on) and ± 1.79% (light‐off), while those at 190–200 s are ± 2.48% (light‐on) and ± 1.86% (light‐off), respectively. For Figure 4h, the average SD values in the initial 0–10 s region are ± 2.56% (light‐on) and ± 1.81% (light‐off), while those at 190–200 s are ± 2.93% (light‐on) and ± 1.87% (light‐off), respectively. (i) Benchmarking the noise‐involved detectivity and response time against state‐of‐the‐art 2D‐material‐based photodetectors.

Explorations of hybrid PMMA/GeSe_2‐x_ based photodetectors under the visible light irradiations with a wavelength of 525 nm are conducted (Figure 4), where the photograph of a fabricated device with paired Ag electrodes are illustrated in the inset of Figure 4c. In Figure 4a, the chopped photoresponse characteristics under the illuminated light intensity of 10 mW/cm^2^ display the rapid response dynamics, where the response time (τ_r_) of 9.6 µs and a decay time (τ_d_) of 6.7 µs are visualized. The markedly fast τ_r_ implicates the underlying extraction of photoexcited carriers must kinetically overwhelm the intrinsic transient delay due to the pronounced carrier capturing/releasing from lattice‐V_Se,_ involved GeSe_2‐x_, which can be mainly attributed to two reasons. First, the drifted carrier mobility is found to be improved (Figure 2d), which facilitates the photoexcited carriers toward the photocurrent contribution. Next, the effective charge separation via the PMMA/GeSe_2‐x_ hybrids (Figure 3e), which fosters the prolonged carrier lifetime and promotes the effective charge extraction. In addition, the temporal response dynamics in viewing τ_r_/τ_d_ of 10.1 µs/8.3 µs, 11.0 µs/10.6 µs, and 13.8 µs/12.3 µs are obtained when the light intensities drop from 8, 6 to 4 mW/cm^2^, respectively, as shown in Figure 4a. The results indicate that the overall response characteristics display reliable charge percolation across the various irradiated light intensities, paving the robust semiconductor nature for practical employment. In addition to τ_r_, the origin of ultra‐low τ_d_ can be elaborated by the combined effects from dual types of trap involvement that synergistically reduce the transit delay. The wavelength‐dependent *I*–*V* measurements are further conducted to clarify the spectral response range of the hybrid PMMA/GeSe_2__−__x_ photodetector. As shown in Figure S20, the device exhibits distinguishable photoresponses under illumination wavelengths from 365 to 740 nm at the same optical power density of 5 mW cm^−^^2^, confirming its broadband photoresponsive capability. Notably, pronounced photocurrent responses are maintained in the visible‐to‐red region, indicating that the vacancy‐engineered and dual‐phase‐regulated GeSe_2__−__x_ nanoparticles enable photoresponse beyond the conventional UV‐detection window of pristine GeSe_2_.

Figure 4b demonstrates the measured characteristics of noise spectral density (NSD) from hybrid PMMA/GeSe_2‐x_ photodetectors, where the bare GeSe_2‐x_ (two‐step treatment) based design is also examined as a reference. The visualized spectrum is implicated by the govern response of flicker noise at low frequency region, that are caused by unavoidable interferences of device response due to defect traps, and frequency‐dependent Johnson–Nyquist thermal noise [57, 58]. By comparing the result from bare GeSe_2‐x_, the explicit reduction of NSD profiles is pronounced at low‐frequency region of the obtained spectrum, where the flicker noise amplitude is markedly reduced by two orders of magnitude, from 3.4 × 10^−10^ A Hz^−1/2^ (GeSe_2‐x_) to 4.5 × 10^−12^ A Hz^−1/2^ (PMMA/GeSe_2‐x_). As indicated in Figure 4b, the effective mitigation of flicker‐noise interference can be attributed to the incorporated PMMA blends with GeSe_2‐x_ nanoparticles, which drives the electrons to quickly fill the trap states of GeSe_2‐x_ and further deactivate the trap‐induced current fluctuations, thus fostering the improved photodetection detectivity by considering the noise contribution. To address the important figure of merits (FOMs) in terms of evaluating signal‐to‐noise ratio, noise equivalent power (NEP) and, normalized photocurrent dark current ratio (NPDR) with respect to the various intensities of irradiated 532 nm lights are displayed in Figure 4c,d, respectively, which are defined as below [59, 60],

(1) $$NEP=\frac{I_{noise}}{R}\;$$

(2) $$NPDR=\frac{R}{I_{noise}}$$

where *I*_noise_ the noise currents, R the photodetection responsivity, and e the electron charge. The essence of the measured NEP spectrum with respect to incident light power shows the overall low NEP amplitude covering the tested intensity levels ranging from 3.6 × 10^−12^ to 8.8 × 10^−12^ WcmHz^−1/2^, which affirms the surpassing of delivered photoresponses relative to the noise fluctuations under the intensity‐modulation of light input. Another indicator of photodetection feasibility, the NDPR spectrum, is also visualized in Figure 4d. We obtain that amplitude of NDPR features the pronounced positive correlation as a function of light‐power level, showing a nonlinear increase of NDPR from 4–10 mW/cm^2^. These characteristics uncover the capacity of light‐power modulation for the employment of light‐triggered multi‐valued logic (MVL). In addition, the measured R and noise‐associated detectivity (D^*^) as a function of light intensity are demonstrated in Figure S21.

To further access the power‐dependent photodetection characteristics, Figure 4e,f presents the dramatically distinct correlations of the measured photocurrents (I_ph_) under high light power density (4–10 mW/cm^2^) and low light power density (0.001‐3 mW/cm^2^), respectively. The expressions can be quantitatively govern by fitting the measured I_photo_ under incident power density of lights (P) via a power‐law dependence,

(3) $$\;I_{photo}\propto P^{a}$$

where α the exponential term responding to the degree of linearity on the photon fluxes with respect to the delivered photocurrents. The fitted α value under light incidence with high power densities is estimated to be 1.95 (Figure 4e), above two‐fold compared with α of 0.84 under the light incidence with low power densities (Figure 4f). It has been noted that α value approaching unity is regarded as a threshold, which represents the nearly ideal diode phenomenon where the absorbed photons are converted to mobile charges. When exceeds the threshold (α= 1), the photoresponses of dual‐trap involved PMMA/GeSe_2‐x_ display the superlinear characteristics, originating from the circumstance where the shallow traps dominate the decay dynamics of photoexcited carriers because the deep traps are rapidly occupied with the photoexcited electrons, coming from the stimulation of large photon fluxes at high light power light levels. Meanwhile, the photogenerated electrons are trapped in shallow trap states that free more holes for remedying the current conduction via prohibiting the carrier recombination, leading to the superlinear rising of photocurrents as light power increases, as shown in Figure 4e. Under the regime of low power density, both of shallow and deep traps participate in the decay dynamics of the photoexcitation phenomenon. In other words, the photoexcited carriers turn to be immobilized by the large density of trap states, resulting in the delayed photoresponses in a form of sublinear photocurrent‐power dependence, as illustrated in Figure 4f. These findings uncover the implications of power‐modulation photodetectors on logical‐circuit employment will be discussed in the next section. The long‐term on/off switchable characteristics are further examined, as presented in Figure 4g,h under high and low power densities of light irradiation, respectively. The stability of on/off‐state currents under 1000 cycles in the ambient condition (1 atm, 25.5 ± 2.8°C, 47.5 ± 3.9% of relative humidity) are visualized, respectively, displaying the reliable and repetitive switches with the data deviation less than 0.6%. Finally, Figure 4i highlights the superior photodetection performance of the hybrid PMMA/GeSe_2‐x_ design compared to state‐of‐the‐art 2D‐material‐based photodetectors [61, 62, 63, 64, 65, 66, 67, 68, 69, 70, 71, 72]. The simultaneous enhancement of response dynamics and detectivity reveals its substantial potential for practical applications. In addition, the detailed measurement conditions from all referenced devices are provided in Table S4.

### 2.5 Origin of Shallow and Deep Traps Based on DFT Validation

**FIGURE 5:**
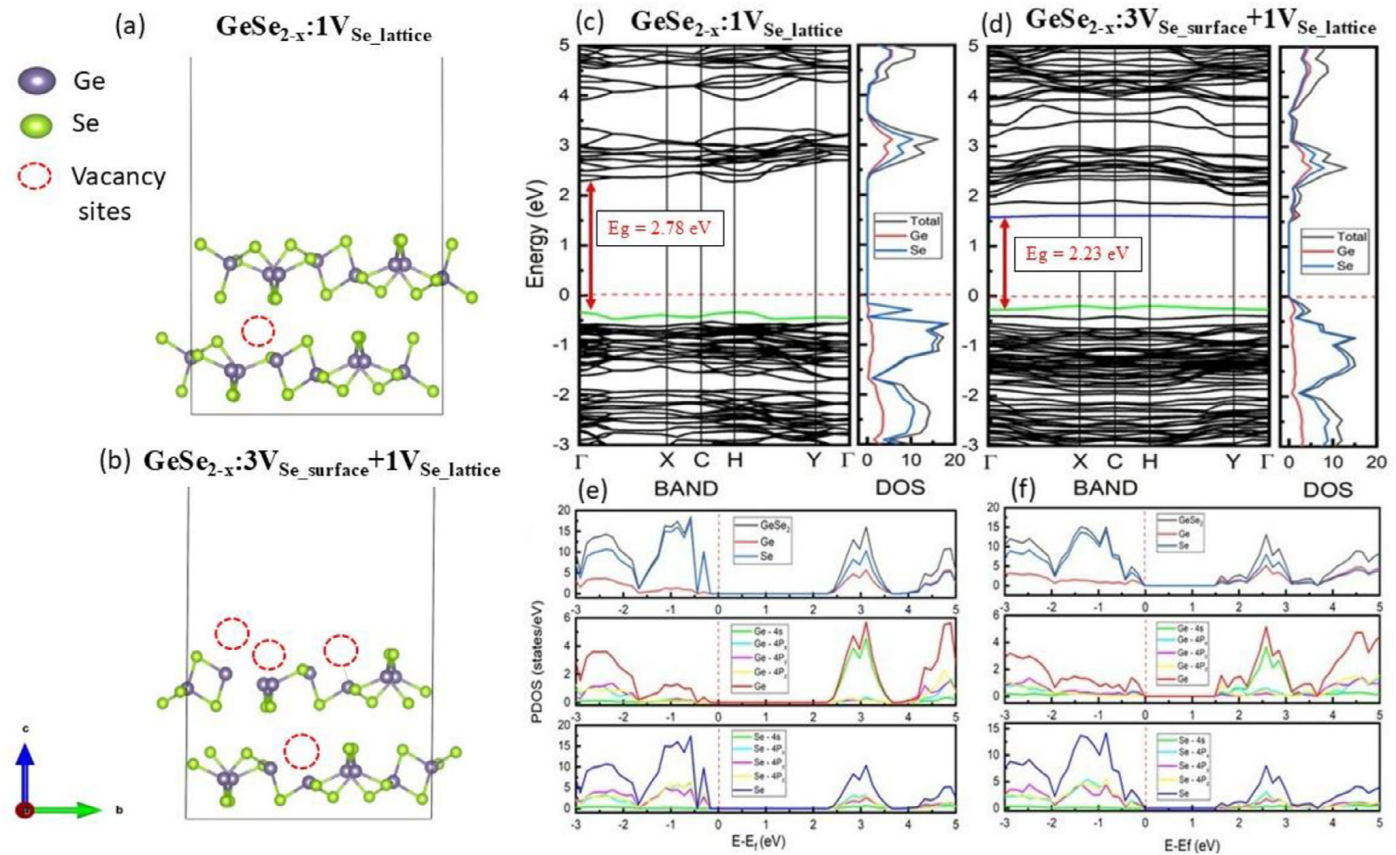
Numerical DFT investigations of Se‐vacancy involved GeSe_2‐x_ lattice: Lattice configurations of (a) GeSe_2‐x_:1V_Se, lattice_, and (b) GeSe_2‐x_:3V_Se, surface_+1V_Se, lattice_, where the calculated band structures along with DOS plots are displayed in (c,d), respectively. Detailed PDOS results of (e) GeSe_2‐x_:1V_Se, lattice_ and (f) GeSe_2‐x_:3V_Se, surface_+1V_Se, lattice_.

To explore the electronic structures of defect‐induced GeSe_2‐x_ structures, vacancy sites, band structure, density of state (DOS), partial density of state (PDOS), and Bader charge analysis via the first‐principles calculations are numerically inspected, as depicted in Figure 5. A primitive cell of β‐GeSe_2_ essentially composes of 16 Ge atoms and 32 Se atoms with the stoichiometric elemental ratio of 2, where two type of V_Se_‐induced GeSe_2‐x_ configurations are examined: The lattice configurations of β‐GeSe_2‐x_ contain an anionic V_Se_ at a lattice site, labeled as GeSe_2‐x_:1V_Se, lattice_ shown in figure above of Figure 5a, and three anionic V_Se_ at surfaces with an anionic V_Se_ in the lattice, labeled as GeSe_2‐x_:3V_Se, surface_+1V_Se, lattice_ shown in figure below of Figure 5a. It should be noted that the chemical bonds of the involved constitutes are generated by the multi‐orbital hybridizations, and the formation energy of V_Se_ is investigated under a wide range of V_Se_ densities to access the thermodynamic considerations, as detailed in Figures S22 and S23. The correlated band structures and DOS characteristics are illustrated in Figure 5c,d, respectively. In the case of GeSe_2‐x_:1V_Se, lattice_, a shallow‐trap energy level at around ‐0.308 eV can be observed near the highest occupied state of valence levels, and the presented E_g_ of 2.78 eV, displaying a bandgap feature, corresponds to the experimental results [one‐step V_Se_ engineered GeSe_2‐x_, Figure 3a]. Furthermore, PDOS results on examining the involved hybridized orbitals point out that such trap evolution is primarily governed by the Se‐4P_z_ orbital. In addition, for the case of GeSe_2‐x_:3V_Se, surface_+1V_Se, lattice_, the E_g_ is reduced to 2.23 eV, which corresponds well to the two‐step V_Se_ engineered GeSe_2‐x_ obtained from the experiments (Figure 3a). These calculated trap levels provide an energetic basis for the experimentally observed dual‐trap behavior. The V_Se, lattice_ configuration generates a shallow trap level close to the valence‐band edge, with an extracted energy of approximately −0.308 eV, whereas the V_Se, surface_ configuration introduces an additional deep trap level at approximately 1.579 eV. Therefore, the calculated trap energies support the assignment of V_Se, lattice_ as the main origin of shallow trap states, and V_Se, surface_ as the origin of deep trap states. This energetic distinction is consistent with the PL/TRPL analyses and the transient photocurrent results, where the shallow and deep traps exhibit different relaxation channels and carrier‐extraction time constants. By calculating the PDOS to access the involved orbital origins, we find that the shallow trap is predominantly caused by the Se – 4P_z_ orbital, while the Se‐4P and Ge‐4P orbitals jointly contribute to the deep‐trap evolution, as shown in Figure 5f. Although the exact structural configurations of fabricated samples remain challenging to be precisely emulated, these findings partially elaborate the distinct modulations of electronic structures obtained from the spatial locations of V_Se_ in GeSe_2‐x_ structures. Another indirect evidence is accessed by probing the charge transfer (∆Q) between V_Se_‐induced trap sites and GeSe_2‐x_ lattices based on Bader charge analysis, where the extracted results are shown in Table 2 [73]. We obtain the value of ∆Q to be ‐0.011e in the case of GeSe_2‐x_:3V_Se, surface_+1V_Se, lattice_, showing a more negative result than that of GeSe_2‐x_:1V_Se, lattice_ (∆Q = ‐0.009e), indicating that the more pronounced electron trapping circumstances are induced in surface‐trap evolution. All these findings elaborate that V_Se, lattice_, and V_Se, surface_ contribute to the shallow‐trap and deep‐trap states, respectively, which significantly influence the photophysical and conduction characteristics of V_Se_‐involved GeSe_2‐x_ lattice system. Additional DFT calculations of Zr‐adsorbed GeSe_2_ models are provided in Figure S24. The calculated band structures and DOS indicate that Zr adsorption does not introduce the formation of defect states near the Fermi level and only marginally affects the band structures, supporting that trace Zr is not the primary origin of the observed shallow/deep trap states.

**TABLE 2:** Numerical results of bandgap energy (E_g_) and charge transfer (Δ*Q*) via Bader charge analysis.

Sample type	E_g_ (eV)	Δ*Q* (e)
GeSe_2‐x_: 1_VSe, lattice_	2.78	−0.009
GeSe_2‐x_:3_VSe, surface_+1_VSe, lattice_	2.23	−0.011

### 2.6 Photonic Multi‐Valued Logic Circuits

Multi‐valued logic (MVL) technology is regarded as a key enabler for next‐generation and data‐intensive digital electronics for the demanding needs of high‐level computations and a wide application window [74, 75]. This contrasts the classical binary‐circuit approach (Boolean logic) based on two fundamental digits, “0” and “1,” because MVL operation relies on logical states constructed by more than three data levels, enabling more compact circuit designs and higher information‐density computation in a single logic gate. By addressing the challenges of circuit‐level development, this new material approach enables seamless integration with Si‐based platforms for MVL realization. It demonstrates practical feasibility and design flexibility, effectively bridging the gap between theoretical layouts and current manufacturing specifications. Diving to these speculations, we corroborate a photonic MVL circuit through the employment of a hybrid PMMA/GeSe_2‐x_ based photodetector with light‐power resolved characteristics. We show the photo‐induced functionality in that their MVL‐circuit operation is triggered by optical excitation of the device channel via the light‐power modulations.

**FIGURE 6:**
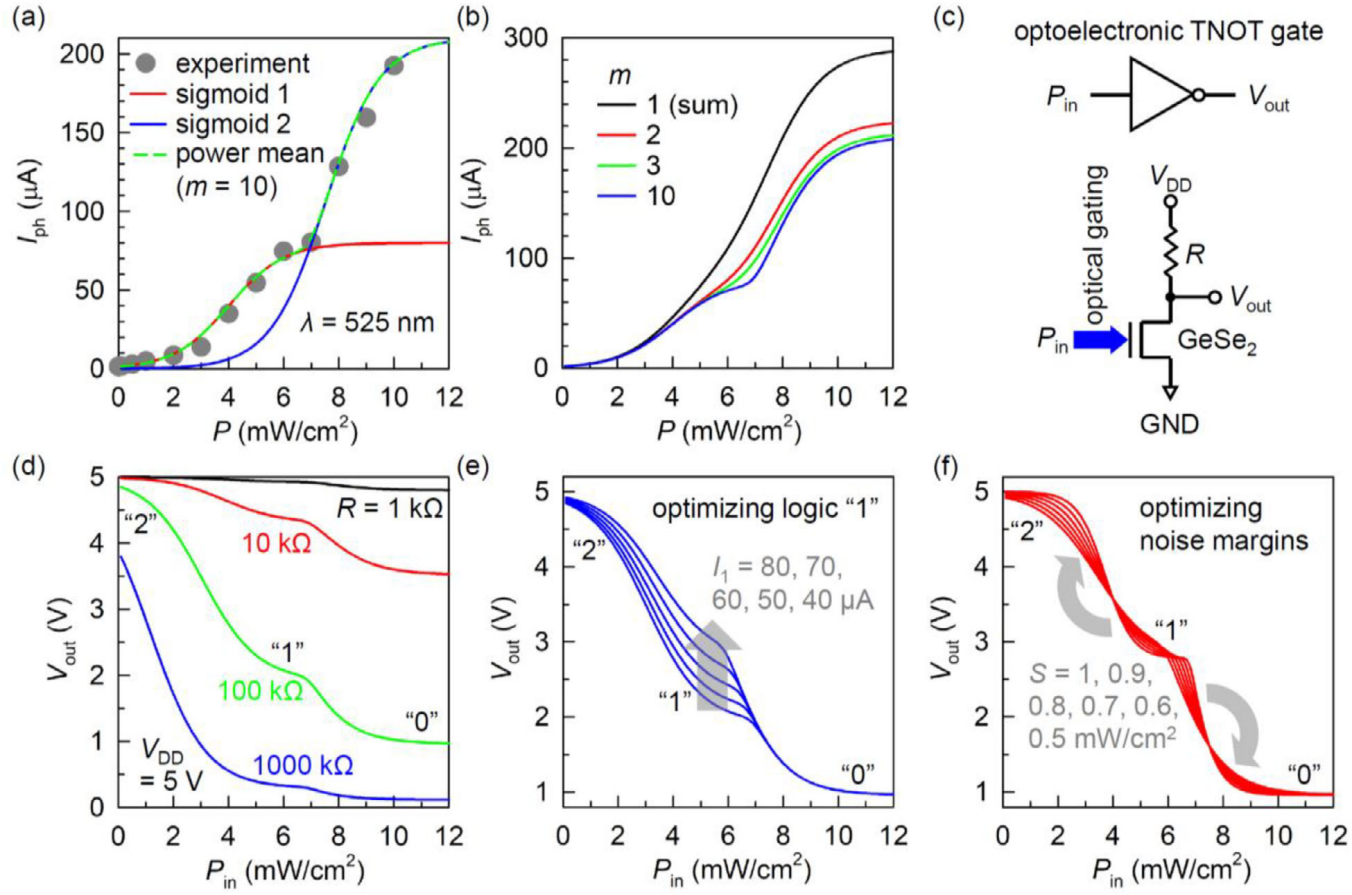
Emerging approaches of MVL implementation based on light‐power resolved characteristics. (a) Correlation between light inputs (wavelength of 525 nm, light power: 0–10 mW/cm^2^) and photocurrent outputs, obtained from experimentally monitored data and analytically fitted results. (b) Numerical modulation of transition parameter, *m*, on *I*_ph_. (c) Schematic illustration of a hybrid optoelectronic circuit for visualizing the logical ternary NOT (TNOT) operation. (d) Fitting of light‐triggered LTC characteristics associated with the value of *R*. Strategies of LTC engineering through the adjustment of (e) *I*_1_ for optimizing logic “1” and (f) *S* parameters for optimizing noise margins into the practical visualization of full‐swing ternary inverter triggered by light‐power modulations.

To model the measured photocurrent–optical power (*I_ph_‐P*) correlation and build it into the photonic logic circuits, the analytic double‐sigmoid functions are introduced to reproduce the major features of the modulated *I_ph_*, governed by the following equations,

(4) $$\sigma_{1}=\frac{I_{1}}{1+\exp\left(-\frac{P-P_{1}}{S}\right)}\;$$

and

(5) $$\sigma_{2}=\frac{I_{2}}{1+\exp\left(-\frac{P-P_{2}}{S}\right)}\;$$

(6) $$I_{ph}={\left(\sigma_{1}^{m}+\sigma_{2}^{m}\right)}^{\frac{1}{m}}\;$$

where *I*_1_ and *I*_2_ are the maximum current levels and correlated *P*_1_ and *P*_2_ are the transition points of the first and second sigmoid functions (*σ*_1_ and *σ*_2_), respectively, *S* is the slope parameter, and *m* the transition parameter. In Equation 6, *I*_ph_ is expressed as a power mean of two sigmoid functions [76]. Figure 6a displays the results of the analytical modeling of *I_ph_‐P* correlations, with rationalized parameters of *I*_1_ = 80 µA, *I*_2_ = 210 µA, *P*_1_ = 4 mW/cm^2^, *P*_2_ = 7.5 mW/cm^2^, *S* = 1 mW/cm^2^, and *m* = 10. The individually calculated *σ*_1_ (sigmoid 1) and *σ*_2_ (sigmoid 2) are incorporated with the govern power‐mean curve as the references, respectively. Modulations of *m* value with respect to the modeling utility are examined in Figure 6b, showing that the algebraic sum of two sigmoid functions (*m* = 1) or low‐order *m* may lose the ternary information about intermediate logic level in between two district states, justifying the essence of higher‐order *m* for modeling the power‐mean characteristics. Figure 6c reveals an internal circuit for an optical ternary NOT (TNOT) gate where a PMMA/GeSe_2‐x_ device serves as a sensitive and stepwise light‐trigger framework in a way to modulate the pull‐up/pull‐down network. Such a circuit system enables to convert a wide range of optical *P*_in_ into an electrical output (*V*_out_) in a feasible route, while performing the logic inversions for ternary “0,” “1,” and “2” levels.

Based on the logical TNOT concept, we build the logic‐transfer curve (LTC) at V_DD_ of 5 V with the examinations of varying *R* values of a series resistor, as displayed in Figure 6d. As *R* increases, the programming pulling‐down strength is progressively increased, directly affecting the swing asymmetry of ternary data levels upon a light‐power domain. Hence, *R* = 100 kΩ of analytic modeling is accessed for balancing *V*_out_ swing and ruling the ideal logic‐inverting performances. Although the essence of optoelectronic TNOT gate is successfully validated in Figure 6d, further steps are still demanded for circuit optimization to minimize the signal distortion. We propose the way by re‐positioning logic “1” underneath the intermediate profiles between discrete “0” and “2” levels via adjusting levels of *I*_1_, to attain the effective pulling of *V*_out_ close to *V*_DD_. Figure 6e confirms this hypothetical strategy, where *I*_1_ = 40 µA is accessed to proceed the next step on minimizing the critics of noise margins in MVL operation. We visualize the results of *S* adjustment ranging from 1 to 0.5 mW/cm^2^, as shown in Figure 6f. We find the virtual potency of *S* adjustment on the critical metric, Static Noise Margin (SNM) of the present TNOT circuit. When gradually reducing *S* parameter from 1.0 to 0.5 mW/cm^2^, the displayed slope of LTC is progressively lifted, representing the improved differentiation efficiency of ternary levels. The institutive specifications may provide more intuitive interpretation: Without the optimization of *S* adjustment (S = 1.0 mW/cm^2^), the light‐power swing (ΔP_in_ is approximately 6.0 mW/cm^2^) is required to attain a logic transition from logic “1” to “0” by switching V_out_ from 5 to 2.8 V; With optimizing the *S* adjustment (S = 0.5 mW/cm^2^), the amended ΔP_in_ is reduced to 4.0 mW/cm^2^ for featuring the transient voltage gain (referring to a slope of transition zone between two discrete levels of LTC) by 1.5‐fold lower than the case of S = 1.0 mW/cm^2^. This explicitly improves the SNM metric of the TNOT circuit by virtue of boosting the stability tolerance of employing high‐density information in logic computation. Further analyses on the level separation margins and the derivatives of the LTC are shown in Figure S25. Overall, a light‐triggered, multi‐stage LTC design on Si chips with a stepwise optimization of analytic treatment is exploited. We present an emerging approach of MVL implementation that allows to adequately amend noise margins in all logic levels, and boosts the MVL‐circuit stability under the employment of high‐density information, which may develop into a potentially useful element for direct logic gates in potentiometric optical sensing and neuromorphic computing.

## 3 Conclusion

We report the dual‐phase GeSe_2‐x_ nanoparticles obtained from two‐step V_Se_ engineering that envision the potential photodetection design, meeting the demanding merits of power‐resolved responsivity, response dynamics, and noise suppression. The origin of partial β‐to‐α phase transformation is mediated by the dual‐defective lattice configurations, manifesting V_Se, lattice_‐induced shallow trap states, and V_Se, surface_‐induced deep trap states. The multifaceted implications are uncovered: (1) We examine the carrier extraction and conduction characteristics under the transient photovoltage, which reflects the involvement of competing trapping mechanisms between low and high‐light‐power photoexcitations. The dual types of traps jointly modulate the frequency response and raise the carrier transport speed at wide operation frequencies. (2) The spectral positions of defect states in GeSe_2‐x_ nanoparticles are experimentally validated via the static and time‐resolved PL characterizations, and the dynamic photophysical phenomena of incorporating a PMMA coating with GeSe_2‐x_ samples visualize the effectiveness of boosting the carrier separation. (3) The origin of phase transformation and trap‐state evolution on band structures is numerically revealed with the DFT calculation. Finally, we corroborate a photonic MVL circuit through the employment of a hybrid PMMA/GeSe_2‐x_ based photodetector. The implementation of effective capability of light‐power modulations at 525 nm in wavelength features the TNOT circuit with the SNM‐metric improvement in all logic levels, paving the way to exploiting the high‐density information and neuromorphic operation in logic computation.

## 4 Experimental Section

### 4.1 Synthesis of Dual‐Phase GeSe_2‐x_ Nanoparticles

Single‐crystalline‐phase GeSe_2_ was grown by the vertical gradient freeze method in a vertical tube furnace. Essentially, stoichiometric mixtures of high‐purity (4N) elemental Ge and Se were sealed in a cone‐shaped quartz ampoule and then heated up to 1073 K for 10 h followed by cooling to 973 K at 1 K/h. Detailed crystallographic and microstructural characterization are presented in Figure S26. For preparing β/α GeSe_2‐x_ nanoparticles, the as‐synthesized GeSe_2_ crystals were subjected to manual grinding for 15 min, and then dispersed in ethanol and ultrasonicated for 6 h to exfoliate the layered structures. The resulting suspension was dried on a ceramic hot plate at 60°C to obtain a preliminary powder. This powder was then further refined by ball milling in a planetary mill at 800 rpm for 5 h. After washing with ethanol and drying again at 60°C, the fine GeSe_2‐x_ nanoparticles dispersed in ethanol were collected for subsequent film fabrication. The photographs of large‐quantity synthesis of samples are presented in Figure S26. Si wafers were diced into 2 cm × 2 cm substrates, and corrosion‐resistant tape was affixed to the backside to prevent damage during subsequent processes. The Si substrates were sequentially cleaned by ultrasonic treatment in acetone, isopropanol, and deionized (DI) water, followed by drying with an air flow. To improve the surface hydrophilicity, the as‐cleaned substrates underwent with standard APM treatment (H_2_O_2_:NH_4_OH:DI = 1:1:5) in a water bath at 82°C for 20 s. The Si substrates were then subjected to a process of metal‐assisted chemical etching to form Si nanorod arrays (SiNRs) on Si substrates (SiNR/Si). Essentially, Si substrates were immersed in a mixed solution containing 0.04 g AgNO_3_ and 4 mL HF in 20 mL DI water for 15 s to form Ag seeds at Si surfaces. After rinsing with DI water, the Ag‐loaded Si substrates were dipped in the etching solutions containing 0.7 mL H_2_O_2_ and 4.2 mL HF, in 20 mL DI water for 60 s. The fabricated SiNR/Si substrates were immersed in 20 mL HNO_3_ for 10 s, and subsequently in diluted HF solutions for 20 s to complete the whole process.

### 4.2 Fabrication of a PMMA/GeSe_2_‐x Hybrid Layer and Device Assembly

1 g of poly(methyl methacrylate) (PMMA, molecule weight of 35 000) was dissolved in N,N‐dimethylformamide (DMF) to form a 1.5 wt.% solution, followed by ultrasonication for 1 h to ensure complete dissolution. Then, 6 mg of GeSe_2‐x_ nanoparticles and 1 µL of a fluorosurfactant (FS‐300) were added to the PMMA solutions and further ultrasonicated for 30 min to obtain a uniform PMMA/GeSe_2‐x_ dispersion. A 200 µL aliquot of composite solutions was drop‐cast onto the SiNR/Si substrates (2 cm × 2 cm), followed by a two‐step annealing process at 60°C for 1 h, and followed by 120°C for 1 h on a ceramic hot plate, to remove the residual solvents and enhance the film quality. Finally, a pair of Ag electrodes was deposited by electron‐beam evaporation (Deposition rate: 1.0 Å s^−^^1^) to form 150 nm thick metal electrodes defined by a shadow mask.

### 4.3 Characterizations

Material characterizations were employed to comprehensively evaluate the structural, compositional, morphological, and optoelectronic properties of the samples. X‐ray diffraction (XRD, Bruker AXS GmbH) was used to identify the crystalline phases, while micro‐Raman spectroscopy (Horiba iHR550, λ = 525 nm) was applied to probe vibrational modes and phase transitions. A synchrotron X‐ray powder diffraction (SXRD) experiment was conducted for phase identification and structure analysis. The SXRD patterns were collected with the MYTHEN detector with a 15 keV beam at beam line 09A, Taiwan Photon Source, National Synchrotron Radiation Research Center (NSRRC) in Hsinchu, Taiwan. High‐resolution transmission electron microscopy (HRTEM, JEOL JEM‐2010) was used to investigate the nanostructures and phase boundaries of a GeSe_2‐x_ nanoparticle. Surface morphology and thickness were further analyzed by atomic force microscopy (AFM, Dimension HPI), scanning electron microscopy (SEM, Hitachi S‐4800), and a surface profilometer (Alpha‐step D‐300). Fourier‐transform infrared spectroscopy (FTIR, JASCO FT/IR 4600) was used to identify the chemical bonds and functional groups, while X‐ray photoelectron spectroscopy (XPS, PHI VersaProbe 4) and ultraviolet photoelectron spectroscopy (UPS, KRATOS ULTRA AXIS DLD) were conducted to analyze the elemental valence states and electronic band structures. The optical properties were characterized by UV–vis spectroscopy (HITACHI U‐3900H). A static PL equipped with a 365 nm LED excitation source, and TRPL spectroscopy with pulsed laser excitation at 430 and 800 nm, respectively, were utilized to characterized the light‐emission behaviors and carrier dynamics, respectively. Electrical and optoelectronic measurements were performed inside a dark box using a semiconductor parameter analyzer (Keysight B1500A). *I*–*V* characteristics under dark and light‐illumination conditions were recorded using LED light sources at various wavelengths (365, 420, 525, 590, 620, and 740 nm), with optical power calibrated using a thermopile power meter (Newport 1919‐R). In addition, a solar simulator with AM 1.5 G source was employed to examine the trap‐associated conduction mechanism, where the signals were collected with a current–voltage measurement system (Keithley 2400). In addition, the ‐3 dB frequency response and NSD characteristics of the photodetector devices were characterized using a commercial photodetector quantum efficiency analyzer (PD‑QE, Enlitech). In addition, the NSD results were recorded over a frequency range from 1–1000 Hz, using a low‑noise current preamplifier and a spectrum analyzer to capture the current noise spectral density across frequency. The integration time and resolution bandwidth were optimized (e.g., 1 s averaging, 1 Hz bandwidth) to suppress random fluctuations and improve measurement fidelity. These conditions ensured reliable extraction of weak signals and enhanced the professionalism of the experimental description.

### 4.4 Transient Optoelectronic Measurements for Trap‐State Analysis

The current–voltage (*I*–*V*) characteristics of the vertical devices were measured under dark conditions to evaluate diode rectification and dark current behavior. For transient photoresponse measurements, the devices were illuminated with a pulsed white light source (AM1.5G equivalent spectrum, covering ∼300–800 nm) at an intensity of 4–10 mW cm^−^^2^. The light was modulated into 100 ms pulses using an electronic shutter, ensuring the devices reached a steady‐state photocurrent before switching off the illumination. Short‐circuit photocurrent decay transients were recorded with a Keysight B1500A semiconductor parameter analyzer. The illumination intensity was tuned by calibrated neutral density filters, and the spectral output of the lamp was confirmed against a standard reference photodiode. The transient photocurrent signals were integrated to determine the photogenerated carrier density under various illumination intensities, providing the basis for analyzing carrier dynamics. In addition, CE measurements were conducted under open‐circuit conditions, where the integration of transient photocurrent yielded the carrier density as a function of open‐circuit voltage. These results were further compared with the ideal semiconductor behavior to analyze the distribution of band‐tail states. Based on the carrier densities obtained from CE measurements, the effective carrier mobility was calculated using a simplified drift‐transport model. The variation of carrier mobility with carrier density was subsequently plotted to elucidate the correlated transport characteristics. For the transient photoresponse measurements, the incident light was modulated using 525 nm with a pulse width of 0.3 ms and a modulation frequency of 0.1–1 kHz. The transient photocurrent signals were recorded using Keysight B1500A semiconductor parameter analyzer under a bias voltage of ‐5 V. The effective bandwidth of the measurement circuit/system was > 100 kHz. The rise time (τ_r_) was extracted as the time required for the photocurrent to increase from 10% to 90% of the steady‐state photocurrent after light illumination, whereas the decay time (τ_d_) was extracted as the time required for the photocurrent to decrease from 90% to 10% after the light was turned off.

### 4.5 Estimation of Figures of Merit for Photodetectors

Multiple performance indexes of photodetectors were employed to comprehensively evaluate their photoelectric conversion capability. Photoresponsivity (R) was defined as the ratio of the photocurrent (I_ph_) to the incident optical power (P_opt_), expressed as below,

(7) $$R=\frac{I_{ph}}{P_{opt}}=\frac{I_{illum.}-I_{dark}}{E_{opt}*a}\;$$

where *I_illum._* the excited currents under light illuminations, *I_dark_* the dark currents, *E_opt_* the incident optical power density measured by a calibrated power meter, and *a* the effective areas under light illuminations. Detectivity (*D^⁎^*) reflected the ability of a photodetector to sense weak optical signals under the influence of noise, expressed as below,

(8) $$D=\frac{R}{\sqrt{2qJ_{d}}}\;$$

where *q* the elementary charge (1.602 × 10^−^^1^ ^9^ C) and *J_d_* the dark current density (A·cm^−^^2^).

### 4.6 First‐Principles Calculations

The DFT calculations were carried out with the Vienna Ab initio Simulation Package (VASP) software, based on the density functional theory. Generalized gradient approximation (GGA) with the employment of Perdew–Burke–Ernzerhof (PBE) was adopted to simulate the exchange–correlation energy for the geometric optimization process [77, 78, 79, 80]. A 5 × 5 × 1 Monkhorst–Pack k‐point grid was employed for sampling the Brillouin zone, with a plane‐wave basis kinetic energy cutoff of 520 eV. Ionic relaxation for all atomic structures was performed with a force convergence criterion of 0.01 eV Å^−^^1^. A large vacuum layer of 15 Å was set to avoid the interaction between layers.

To determine the most stable configuration of a Se‐vacancy involved in GeSe_2_, the formation energy (*E_f_*) was employed, representing the energy required to form the defects by removing a Se atom from the crystal lattice. The formation energy (*E_f_*) was calculated using the following equation:

(9) $$E_{f}=E_{\mathrm{GeSe}2:V_{Se}}+n_{Se}\mu_{Se}-E_{\mathrm{GeSe}2}$$

where $E_{\mathrm{GeSe}2:V_{Se}}$ the total energy of the system with selenium vacancies, *n_Se_* the number of Se atoms removed to create the vacant defect. µ_*Se*_ the chemical potential of Se, derived from bulk Se as $\mu_{Se}=\frac{E_{Se,bulk}}{N_{Se}}\;$ (*E*_*Se*,*bulk*_ and *N_Se_* represent the total energy and number of Se atoms of a unit cell, respectively. Accordingly, the calculated chemical potential of Se atoms (µ_*Se*_) was ‐3.5038687 eV. Moreover, the total energy of the pristine GeSe_2_ lattice, given as *E*_GeSe2_ was ‐197.28411 eV. In addition, the charge transfer was assessed using numerical Bader charge analysis.

### 4.7 Simulation and Modeling of MVL Circuits

Representative *I_ph_*–*P* data measured on the GeSe_2_ photodetector at *λ* = 525 nm were taken as an input because of their pronounced multiple‐regime responses associated with different structural and energetic origins of charge trap states. Based on the dual‐sigmoid function fitting of these input data, the values of *I_ph_* were converted to equivalent device resistances at different incoming optical powers. The circuit‐level simulation of photonic MVL gates was performed by using the voltage divider set by the optically controlled GeSe_2_ device resistance and a fixed series resistance. This procedure was used to find the optimum values of both internal (*I*_1_ and *S*) and external (*R*) parameters that significantly affect the optically modulated LTC.

### 4.8 Statistical Analysis

In this study, to evaluate the repeatability and statistical reliability of the electrical and optoelectronic characteristics, 12 independently fabricated devices were prepared for each electrical and photoelectrical measurement. The parameters presented in Figure 2d–i and Figure 4c–h were statistically analyzed based on measurements obtained from these 12 devices. Specifically, the statistical analysis was performed for the effective charge carrier mobility as a function of carrier concentration derived from charge extraction measurements (Figure 2d), the normalized carrier density versus open‐circuit voltage relationship obtained from the integration of charge extraction transients (Figure 2e), and the ‐3 dB cutoff frequency responses in the low‐ and high‐frequency regimes for one‐step and two‐steps V_Se_‐engineered GeSe_2‐x_ photodetectors (Figure 2f–i). In addition, the statistical evaluation was also carried out for the NEP and NPDR characteristics (Figure 4c,d), the power‐resolved photoresponses under high and low light power densities (Figure 4e,f), and the long‐term light on/off switching stability measurements under high‐ and low‐intensity illumination conditions (Figure 4g,h).

## Author Contributions

**Chia‐Nung Kuo**: investigation, data curation, validation. **Pin‐Chao Liao**: conceptualization, investigation, validation, visualization, writing – original draft, methodology. **Chun‐Jen Wang**: conceptualization, validation, visualization, investigation, writing – original draft, writing – review and editing, data curation. **Chin Shan Lue**: supervision, resources, investigation. **Chia‐Yun Chen**: conceptualization, writing – original draft, writing – review and editing, supervision, resources, methodology. **An‐Ting Tsai**: conceptualization, investigation, validation, visualization, data curation, writing – original draft, writing – review and editing. **Po‐Hsuan Hsiao**: writing – original draft, investigation, visualization, validation. **Chang‐Hyun Kim**: investigation, writing – original draft, writing – review and editing, data curation, supervision, methodology. **Le Vo Phuong Thuan**: software, data curation, validation, investigation.

## Conflicts of Interest

The authors declare no conflicts of interest.

## Supporting information




**Supporting File**: advs76324‐sup‐0001‐SuppMat.docx.

## Data Availability

The data that support the findings of this study are available from the corresponding author upon reasonable request.
