# Supplementary material for: Intrinsic Dual‐Phase Regulated GeSe2 Nanoparticles Triggered by Ball‐Milling Treatment for Photonic Multi‐Valued Logic Circuits
Source: Adv Sci (Weinh). 2026 Jul 1;13(53):e76324. doi: 10.1002/advs.76324 (PMC13337021; doi:10.1002/advs.76324)
Supplement: Supplementary file 1 [file ADVS-13-e76324-s001.docx]

Supporting Information

**Intrinsic dual-phase regulated GeSe₂ nanoparticles triggered by ball-milling treatment for photonic multi-valued logic circuits**

*An-Ting Tsai, Chun-Jen Wang, Pin-Chao Liao, Chin Shan Lue, Chia‐Nung Kuo, Le Vo Phuong Thuan, Po-Hsuan Hsiao, Chang-Hyun Kim*, and Chia-Yun Chen**

**S1 Structural evolution of GeSe_2-x_**

**S1-1. XRD analysis of GeSe₂-x nanoparticle**s **obtained from different ball-milling durations**

**Figure S1.** XRD patterns of obtained GeSe_2-x_ nanoparticles after undergoing solid-state ball milling for 1, 3, and 5 h, respectively. The crystallgraphic β-phase reflections at (002), (004), and (006) are gradually suppressed with the prolonged ball-milling treatment, while additional α-phase peaks, including (15̅1), (180), and (2̅53), can be found.

**S1-2 Raman spectra of bulk and exfoliated GeSe₂**

**Figure S2.** Raman spectra of bulk GeSe₂ crystals and exfoliated GeSe₂ flakes. Both samples exhibit the characteristics of β-phase vibrational mode centered at 210 cm⁻¹. Compared with the result of bulk GeSe₂ crystals, the Raman features at 210 cm⁻¹ of exfoliated flakes display the relatively sharper peaks with slightly enhanced intensity, reflecting the improved crystallinity and reduced phonon scattering.

**S2 Compositional analysis**

**S2-1 ICP analysis of GeSe_2-x_ nanoparticles treated with one-step and two-steps V_Se_ engineering**

**Figure S3.** Quantitative examinations extracted from ICP measurements of GeSe**_2-x_** nanoparticles treated with (a) one-step and (b) two-steps V_Se_ engineering, revealing a atomic ratios of Ge:Se to be 1:1.98 and 1:1.6, respectively.

**S2-2 EDS spectra of GeSe_2-x_ nanoparticles treated with one-step and two-steps V_Se_ engineering**

**Figure S4.** Compositional EDS analysis of GeSe**_2-x_** nanoparticles treated with (a) one-step and (b) two-steps V_Se_ engineering, revealing a atomic ratios of Ge:Se to be 1:1.98 and 1:1.61, respectively.

**S3 Surface characterizations of GeSe_2-x_ nanoparticles treated with one-step and two-steps V_Se_ engineering**

**S3-1 XPS spectra**

**Figure S5.** Ge 2p XPS spectra of (a) one-step V_Se_ engineered, (b) two-steps V_Se_ engineered GeSe**_2-x_** nanoparticles, (c) hybrid PMMA/GeSe**_2-x_** treated with two-steps process and (d) XPS elemental-ratio analysis. The atomic ratio of Ge:Se estimated from the oxidation state in Figure S5(a) is estimated to be 1:2, and the relatively qunatitative results estimated from the integration of characteristic-peak area in Figures S5(b) and S5(c) are found to be appriximately similar (1:1.64 and 1:1.67, respectively). These results indicate that after ball-milling treatment, the Se atoms lose the full coordination with Ge lattices and reflect multiple oxidation states (Ge^2+^ and Ge^4+^) for sustaining the chemical and charge balances. The evolution of Se vacancies is accompanied with the emergence of Ge²⁺ states, which further confirms that electron accumulation occurs on Ge atoms due to Se deficiency. With PMMA encapsulation [Figure S5(c)], the XPS intensity of Ge²⁺ is sightly reduced, suggesting that PMMA coating may mitigate the portion of surface defects and enhance the chemical stability of treated GeSe_2-x_ nanoparticles.

**S4 EPR analysis**

**Figure S6.** EPR spectra of bulk GeSe₂ (black) and GeSe**_2-x_** nanoparticles treated with one-step (blue) and two-steps (red) V_Se_ engineering . A distinct symmetric signal at g = 2.003 is envisioned, corresponding to the paramagnetic defect states associated with Se deficiency.

**S5 Stastatic evalution of size dstributions**

**Figure S7.** Size distributions of the synthesized GeSe**_2-x_** nanoparticles treated with two-steps V_se_ engineering, where the average size of GeSe**_2-x_** nanoparticles estimated from the TEM observation [Figure 1c in the main text] is 12.9 nm with a standard deviation (SD) of 2.54 nm.

**Figure S8.** To ensure the fabrication reproducibility, the samples were re-fabricated following the similar protocol. Representative TEM images of GeSe**_2-x_** nanoparticles obtained from a (a) second run and (b) third run. The correlated size distributions in a (c) second run and (d) third run. The average sizes (standard deviation) of samples fabricated in a second run and third run are 13.3 nm (2.78 nm) and 12.8 nm (3.05 nm), respectively, showing the sound controllability and reproducibility.

**S6 Additional phase-identification and semi-quantitative analysis of β/α GeSe₂₋ₓ nanoparticles**

**Figure S9.** Additional SAED evidence for the coexistence of β- and α-phase domains in ball-milled GeSe₂₋ₓ nanoparticles. (a) Representative low-magnification TEM image of the nanoparticle region used for SAED analysis. (b) Corresponding SAED pattern collected from the ball-milled GeSe₂₋ₓ nanoparticles. (c) Indexed SAED pattern showing β-GeSe₂-related diffraction features together with the α-GeSe₂-related diffraction feature at d ≈ 0.267 nm, assigned to the α-GeSe₂ (180) plane. These results further support the coexistence of crystalline β- and α-phase domains after the two-step V_Se_-engineering and ball-milling treatment, rather than complete amorphization.

**Figure S10.** Raman peak-deconvolution analysis of ball-milled GeSe₂₋ₓ nanoparticles for semi-quantitative evaluation of β/α phase contributions. (a) Representative Raman spectrum showing the β-GeSe₂-related vibrational feature around 210 cm⁻¹ and the α-GeSe₂-related vibrational feature around 198 cm⁻¹. (b–d) Peak-deconvolution results obtained from representative spectra of the ball-milled GeSe₂₋ₓ nanoparticles. The fitted peak areas were used for semi-quantitative comparison of the relative β- and α-phase-related contributions.

**Table S1.** Semi-quantitative comparison of β- and α-phase-related Raman peak contributions extracted from the peak-deconvolution analysis of ball-milled GeSe₂₋ₓ nanoparticles.

| Sample | A_α_ | A_β_ | A_α_ /A_β_ |
| --- | --- | --- | --- |
| one-step treatment | 89170.91 | 135991.6815 | 0.66 |
| Two-steps treatment | 161145.12 | 40371.74 | 3.99 |
| Two-steps treatment+PMMA | 93661.20 | 28043.75 | 3.34 |

To further verify that the α-phase-related Raman feature does not originate solely from the amorphous or glassy GeSe₂, the additional SAED and Raman peak-deconvolution analyses are performed. The indexed SAED pattern exhibits the distinguishable diffraction rings/spots associated with crystalline β-GeSe₂- and α-GeSe₂-related domains, indicating that ordered lattice features are retained after ball milling. Therefore, although local surface disorder may exist due to mechanical milling, the structural evidence supports β/α crystalline domain coexistence rather than complete mechanochemical amorphization. The extracted Raman peak-area ratio of the PMMA/GeSe₂₋ₓ sample shows A_α_/A_β_ = 3.34, indicating that the α-phase-related contribution is still well preserved after PMMA incorporation. This result suggests that PMMA encapsulation does not significantly suppress the β/α dual-phase configuration of the ball-milled GeSe₂₋ₓ nanoparticles.

**S7 Dark I–V behavior of hybrid PMMA/GeSe_2-x_**

**Figure S11.** (a) I-V characteristics of hybrid PMMA/GeSe_2-x_, treated with different ball-milling durations, including 1 hr, 3 hr, and 5 hr, on SiNR/Si substrates under dark condition. (b) I-V characteristics of hybrid PMMA/GeSe_2-x_ on bare Si and PMMA/GeSe_2-x_ on SiNR/Si substrates under dark condition, respectively.

**S8 SCLC-like trap-limited conduction analysis of hybrid PMMA/GeSe₂₋ₓ photodetectors**

**Figure S12.** log(I)–log(V) characteristics of the hybrid PMMA/GeSe₂₋ₓ photodetector measured under dark conditions and under different light-power densities, respectively. The curves were replotted from the I–V characteristics to evaluate the trap-limited conduction behavior. Under the dark and low-power illumination conditions, the log(I)–log(V) profiles exhibit the SCLC-like trap-limited conduction features, including an ohmic region with a slope close to unity and a trap-filling region with a slope larger than 2. Under the high-power illuminations, the profiles deviate from the conventional voltage-controlled SCLC trend, indicating that photogenerated carriers progressively fill the trap states and promote the light-assisted carrier extractions.

**Table S2.** Fitted slopes extracted from the log(I)–log(V) characteristics of the hybrid PMMA/GeSe₂₋ₓ photodetector under dark and different light-power densities, respectively. The slope values are used to distinguish the ohmic, trap-limited SCLC-like, trap-filling, and photoconductive drift-assisted transport regimes.

| **Illumination power** | **Low-voltage slope**  **( 1V~3V)_** | **Intermediate-voltage slope**  **(3V~5V)** | **High-voltage slope** | **Assigned regime** |
| --- | --- | --- | --- | --- |
| dark | ~1 | >2 | trap-filled | trap-limited SCLC |
| 0.01 mW cm⁻² | ~1 | >2 | trap-filled | trap-limited SCLC |
| 0.1 mW cm⁻² | ~1 | >2 | trap-filled | trap-limited SCLC |
| 5 mW cm⁻² | nonlinear / light-assisted | — | — | trap-filled photoconductive |
| 10 mW cm⁻² | nonlinear / light-assisted | — | — | trap-filled photoconductive |

**S9 Carrier dynamics and trap-state phenomena**

**S9-1 Photocurrent decay dynamics of synthesized GeSe_2-x_ nanoparticles treated with one-step V_se_ engineering under various sonication durations**

**Figure S13.** Photocurrent decay dynamics of synthesized GeSe**_2-x_** nanoparticles treated with one-step V_se_ engineering under various sonication durations, including (a) 1.5 hr, (b) 3 hr, and (c) 6 hr. The decay time constants increase with prolonged treatment durations, suggesting the enhanced shallow-trap-assisted carrier capture–release cycles that extend carrier lifetime but slow recovery features.

**S9-2 Optical absorption and operational stability of PMMA-assisted GeSe₂₋ₓ photodetectors**

**Figure S14.** Photocurrent decay transients of two-steps V_Se_ engineered GeSe_2-x_ with and without PMMA encapsulation under 10 mW/cm² of light illuminations. (a) Hybrid PMMA/GeSe_2-x_ with τ ≈ 20 μs and (b) bare GeSe_2-x_ with τ ≈ 60 μs.

**Figure S15.** UV–visible absorption spectra of PMMA coating, two-steps engineered GeSe₂₋ₓ, and hybrid PMMA/GeSe₂₋ₓ samples.

**Figure S16.** Operational stability results of GeSe₂₋ₓ photodetectors with and without PMMA encapsulation under repeated/continuous 525 nm illumination.

To clarify whether PMMA acts as an optical absorption-enhancement layer or mainly as a passivation/encapsulation component, UV–visible absorption and operational stability measurements were performed, as shown in Figures S15 and S16. The absorption spectra show that the light-absorption contribution from the PMMA coating is negligible at the wavelength of 525 nm. Therefore, the improved photoresponse cannot be mainly attributed to the optical absorption enhancement from the PMMA coating. Instead, the operational stability comparison shows that the photocurrent decay after 200 s is reduced from 11.4% for the bare GeSe₂₋ₓ device to 3.7% for the hybrid PMMA/GeSe₂₋ₓ device, with the standard deviation decreasing from 4.3% to 0.11%. These results support that the PMMA coating primarily contributes to surface/interface passivation and encapsulation-assisted stability.

**S10 Frequency response of hybrid PMMA/GeSe_2-x_ based photodetectors**

**Table S3.** –3 dB cutoff frequency of hybrid PMMA/GeSe_2-x_ based photodetectors **(**two-steps V_Se_ engineering) under various illumination power densities. At higher light intensities, the cutoff frequency increases significantly, reaching 31.74 kHz at 10 mW/cm². This trend indicates trap filling under strong illumination, which accelerates carrier transport and improves response speed.

| Power Density  (mW/cm^2^) | Cut-off frequency  (kHz) |
| --- | --- |
| 10 | 31.74 |
| 8 | 29.93 |
| 6 | 16.14 |
| 4 | 10.54 |
| 2 | 8.80 |
| 1 | 7.64 |
| 0.5 | 4.50 |

**S11 Measured optical bandgap Evolution of various treated GeSe_2-x_**

**S11-1 UV-Vis absorption measurements of GeSe_2-x_ via diifferent treatments**

**Figure S17.** UV-Vis spectra of bulk GeSe_2_ and GeSe_2-x_ under different treatments.

**S11-2 UV–Vis light-absorption spectra of GeSe_2-x_ treated with various sonication durations**

**Figure S18.** UV–Vis spectra and Tauc plots of synthesized one-step V_Se_ engineered GeSe_2-x_ under various sonication durations (1.5-6 hr), respectively. These allow the adjust the resulting E_g_ values in the range of 2.52–2.74 eV, respectively.

**S12 UPS spectra of GeSe_2-x_ treated with one-step and two-steps V_Se_ engineering**

**Figure S19.** UPS analyses of (a) two-steps treated GeSe_2-x_ and (b) PMMA/GeSe_2-x_. The measured Fermi levels are estimated to be 4.32 eV and 5.16 eV, respectively.

**S13 Wavelength-dependent photoresponse of hybrid PMMA/GeSe₂₋ₓ photodetectors**

**Figure S20.** Wavelength-dependent I–V characteristics of the hybrid PMMA/GeSe₂₋ₓ photodetector measured under dark conditions and under illumination wavelengths of 365, 420, 525, 590, 620, 660, and 740 nm at an optical power density of 5 mW cm⁻², respectively.

**S14 Measured responsivity and detectivity of hybrid PMMA/GeSe_2-x_ based photodetectors**

**Figure S21.** Photodetection characteristics of hybrid PMMA/GeSe_2-x_ based photodetectors: Responsivity under a range of (a) low light powers and (b) high light powers, respecitvely. Detectivity under a range of (c) low light powers and (d) high light powers, respecitvely. The average SD values are ±2.64 % for Figures S21(a) and 21(b) and ±3.43 % for Figures S21(c) and S21(d).

**Table S4. Comprehensive benchmark of device performance and specific measurement conditions against state-of-the-art 2D-material-based photodetectors.**

| **Device** | **Wavelength** | **Bias** | **Responsivity** | **Detectivity(Jones)** | **Rise/Fall Time** | **Ref.** |
| --- | --- | --- | --- | --- | --- | --- |
| PdSe_2_ | 532~1064 nm | 0 V | 53 mA/W | 5.17 × 10^11^ | 0.72 / 0.24 ms | [53] |
| TiO_2_/WS_2_ | 300~400 nm | 1 V | 1.2 A/W | 1.1 × 10^11^ | - / 14.4 s | [54] |
| Bi_2_O_2_Se | 365 nm | 0 V | 10.93 mA/W | 2.45 × 10^8^ | 9 / 12 ms | [55] |
| InSe | 300~1000 nm | 0 V | 0.103 A/W | 1.83 × 10^10^ | 1 ms | [56] |
| β-In_2_Se_3_ | 898 nm | -8 V | 3 mA/W | 1 × 10^9^ | - / 7 ms | [57] |
| h-BN | 212 nm | 20 V | 0.1 mA/W | 2.4 × 10^8^ | 0.32 / 0.63 s | [58] |
| MoSSe | 375~1550 nm | 1 V | 15 mA/W | 6.91 × 10^9^ | 43 / 38 ms | [59] |
| MoTe_2_ | 637 nm | 10 V | 50 mA/W | 3.1 × 10^9^ | 1.6 / 1.3 ms | [60] |
| WSe_2_ | 980 nm | 0 V | 689.8 mA/W | 1.59 × 10^13^ | 12 / 30 μs | [61] |
| MoSe_2_ | 470 nm | -15 V | 1615 mA/W | 5.05 × 10^11^ | 2.3 / 2 ms | [62] |
| Bi_2_Se_3_ | 735 nm | 0.1 V | 10.1 mA/W | 4.63 × 10^8^ | 37 /62 ms | [63] |
| SnSe/Si | 1064 nm | 0 V | 54.7 mA/W | 7.87 × 10^10^ | 26 / 47 ms | [64] |
| PMMA/GeSe_2-x_ | 365~740 nm | -5 V | 30 mA/W | 7.57 × 10^11^ | 9.63 / 6.7 μs | This work |

**S15 Supplementary DFT results**

**S15-1 DFT calculations of pure β-GeSe_2_ crystals**

**Figure S22.** Details of the β-phase GeSe_2_ lattice configurations and calculated band information. (a) Geometric structure and (b) correlated elemental charge density. (c) Calculated band diagrams and correlated density of states and (d) partial density of states.

This configuration of β-GeSe_2_ possesses a monoclinic structure with lattice parameters of a = 7.016 Å, b = 16.796 Å, c = 11.831 Å, and β = 90.65°. The electronic characteristics of GeSe_2-x_ are examined by calculating the charge density, band structure, density of states (DOS), and partial density of states (PDOS), as shown in Figure S22. The geometric structures and elemental charge density of β-GeSe_2_ are displayed in Figures S22(a) and S22(b), respectively. The band diagram of the β-GeSe₂ features a direct semiconductor property with the bandgap energy of 2.32 eV at the Γ point [Figure S22(c)], as determined using the HSE06 hybrid exchange-correlation functional, which presents good accuracy in electronic structure calculations compared to standard functionals like GGA-PBE, which gives a band gap of 1.34 eV for β-GeSe₂, respectively. According to DOS and PDOS calculations, the valence band energy states in β-GeSe₂ are primarily contributed by Se atoms, whereas the conduction band energy states arise from contributions of both Ge and Se atoms, as illustrated in Figure S22(d). These findings reveal that the covalent nature of the Ge-Se bonds, as evidenced by charge-density overlap [Figure S22(b)], supports the sp³ hybridization model, which aligns with the near-tetrahedral coordination observed in the crystalline structures of β-GeSe₂.

**S15-2 Formation energy of Se vacancies in layered β-GeSe₂**

**Figure S23.** Dependence of formation energy and concentration of (a) bilayer GeSe_2-x_, (b) tetralayer GeSe_2-x_.

Formation energy of V_Se_ in GeSe_2_ lattices serving as a thermodynamic indicator to assess the vacancy formation and structural stability of GeSe_2-x_. These are examined by monitoring the quantities of formation energy with respect to the density of V_Se_ ranging from 2.5% to 34.0%, under the considerations of bilayer GeSe_2-x_ and tetralayer GeSe_2-x_ lattices, as displayed in Figures S22(a) and S22(b), respectively. Spatial configurations of V_Se_ are probed at three various sites: inner lattice sites, surface sites and both lattice and surface sites, respectively. The calculated results indicate that the defect-induced GeSe_2-x_ remains to be thermodynamically stable under the existence of low density of V_Se_ within bilayer and tetralayer GeSe_2-x_ structures.

**S16 DFT calcultion of Zr adsorbed-GeSe₂ monolayers**

**Figure S24.** Geometric structures, electronic band structures, and density of states (DOS) of Zr-adsorbed GeSe₂ monolayers. (a) Optimized geometric structure of a GeSe₂ monolayer with a single Zr atom adsorbed. (b) Optimized geometric structure of a GeSe₂ monolayer with four Zr atoms adsorbed. (c) Electronic band structure and density of states of the GeSe₂ monolayer with one adsorbed Zr atom. (d) Electronic band structure and density of states of the GeSe₂ monolayer with four adsorbed Zr atoms.

**S17 Quantitative analysis of photonic ternary NOT logic operation**

**Figure S25.** Quantitative analysis on the photonic MVL circuit. (a) Dependence of the output level separation margins on *I*_1_. The output “0,” “1,” and “2” voltages at each *I*_1_ were extracted from Figure 6 (e) at *P*_in_ of 0.1, 6, and 12 mW/cm^2^, respectively. The margin between “0” and “1” (*M*_01_) and that between “1” and “2” (*M*_12_) were calculated using these voltages. 2 V is suggested as an ideal margin (for both *M*_01_ and *M*_12_) as it marks the middle point between the total variation (between 1 and 5 V). The lowest *I*_1_ yields the most balanced separations. (b) |*dV*_out_/*dP*_in_| calculated from Figure 6 (f) at different *S* values. Decreasing *S* improves both the optical-to-electrical gain at transition points and the stability of *V*_out_ against fluctuation in *P*_in_ at normal input points.

**S18 Characterizations of bulk samples**

**Figure S26.** (a) Micrographs of dual-phase GeSe_2-x_ nanoparticles at each fabrication stage (From left to right): As synthesized bulk GeSe_₂_ crystals 🡪 Samples under manual grinding for 15 min 🡪 Samples subjected to ultrasonication for 6 hr (One-step V_se_ engineered treatment)🡪 Samples subjected to solid-state ball milling for 5 hr (Two-steps V_se_ engineered treatment). (b) Single-crystal XRD (SXRD) results of bulk, single-crystal GeSe_₂_ (λ = 0.82656 Å) and (c) correlated SXRD along the c axis. The insets show the Laue diffraction pattern along the c axis. (d) HRTEM images of bulk GeSe_₂_ crystals and (e) correlated EDS mapping results. (f) Micrographs of as-prepared GeSe_₂-x_ dispersed in DMF solutions via one-step V_se_ engineered treatment (figure left) and two-steps V_se_ engineered treatment (figure right).
